# Barriers of Influenza Vaccination Intention and Behavior – A Systematic Review of Influenza Vaccine Hesitancy, 2005 – 2016

**DOI:** 10.1371/journal.pone.0170550

**Published:** 2017-01-26

**Authors:** Philipp Schmid, Dorothee Rauber, Cornelia Betsch, Gianni Lidolt, Marie-Luisa Denker

**Affiliations:** 1 Center for Empirical Research in Economics and Behavioral Sciences, University of Erfurt, Erfurt, Germany; 2 Department of Media and Communication Sciences, University of Erfurt, Erfurt, Germany; University of Hong Kong, HONG KONG

## Abstract

**Background:**

Influenza vaccine hesitancy is a significant threat to global efforts to reduce the burden of seasonal and pandemic influenza. Potential barriers of influenza vaccination need to be identified to inform interventions to raise awareness, influenza vaccine acceptance and uptake.

**Objective:**

This review aims to (1) identify relevant studies and extract individual barriers of seasonal and pandemic influenza vaccination for risk groups and the general public; and (2) map knowledge gaps in understanding influenza vaccine hesitancy to derive directions for further research and inform interventions in this area.

**Methods:**

Thirteen databases covering the areas of Medicine, Bioscience, Psychology, Sociology and Public Health were searched for peer-reviewed articles published between the years 2005 and 2016. Following the PRISMA approach, 470 articles were selected and analyzed for significant barriers to influenza vaccine uptake or intention. The barriers for different risk groups and flu types were clustered according to a conceptual framework based on the Theory of Planned Behavior and discussed using the 4C model of reasons for non-vaccination.

**Results:**

Most studies were conducted in the American and European region. Health care personnel (HCP) and the general public were the most studied populations, while parental decisions for children at high risk were under-represented. This study also identifies understudied concepts. A lack of confidence, inconvenience, calculation and complacency were identified to different extents as barriers to influenza vaccine uptake in risk groups.

**Conclusion:**

Many different psychological, contextual, sociodemographic and physical barriers that are specific to certain risk groups were identified. While most sociodemographic and physical variables may be significantly related to influenza vaccine hesitancy, they cannot be used to explain its emergence or intensity. Psychological determinants were meaningfully related to uptake and should therefore be measured in a valid and comparable way. A compendium of measurements for future use is suggested as supporting information.

## Introduction

Influenza is a significant health threat in our world today. For instance, seasonal influenza alone leads to an estimated 3 to 5 million cases of severe illness, and about 250,000 to 500,000 deaths globally each year [1]. Most deaths associated with influenza occur among the most vulnerable members of the world population, i.e. very young children, the elderly and chronically ill patients. Despite influenza’s severity and the availability of safe vaccines, low influenza vaccine uptake rates within specific risk groups remain a challenge throughout the globe and contribute to the burden of disease [2]. The scope of the issue became particularly clear during the 2009–2010 H1N1 pandemic [3]. Vaccine uptake in the general public was very low, with countries reporting less than 50% of the expected coverage in target populations all over the globe ([4]; *Europe*: [5] *China*: [6]; *Australia*: [7]; *USA*: [8]). Even more worrying is the fact that vaccine uptake in high risk groups, such as pregnant women [7] and the elderly [9], were similarly low.

### Influenza vaccine hesitancy

In recent years, several researchers have focused on identifying potential barriers to vaccine acceptance [10–12]. Foremost, the WHO SAGE working group defined the concept of *vaccine hesitancy*. Vaccine hesitancy describes the acceptance of vaccines on a continuum between demand and no demand ranging from accepting all vaccines to accepting no vaccine [13]. According to their work, “vaccine hesitancy refers to a delay in acceptance or refusal of vaccination despite availability of vaccination services. Vaccine hesitancy is complex and context specific, varying across time, place, and vaccines” [14]. This definition of vaccine hesitancy suggests that barriers to vaccine uptake can be very different in kind and significance, with regard to the vaccine and disease in focus. Influenza vaccines, as compared to other standard vaccines, have some special characteristics that should also be considered when looking at influenza vaccine hesitancy. Namely, vaccine effectiveness varies annually and is frequently low [14,15]. Vaccination is required annually; in most countries it is recommended for specific risk groups only and there are influenza-specific myths (e.g. the flu shot can cause the flu [16]). Thus, influenza vaccine hesitancy has unique features that justify further investigations in order to gain a specific understanding of the phenomenon.

Previous systematic reviews on predictors of influenza vaccine uptake within certain risk groups provide initial insights to understand influenza vaccine hesitancy (pregnant women [17]; elderly [18]; healthcare personnel [19,20]; general public [21]). However, different methodologies regarding the search strategies and literature selection criteria complicate comparisons and synthesis of results between risk groups. Moreover, potential differences between predictors of pandemic and seasonal influenza within one risk group are, except for pregnant women [17], not addressed by previous reviews. Lastly, and to the best of the authors’ knowledge, systematic reviews on influenza vaccine hesitancy for children and chronically ill individuals do not exist. The present systematic review bridges the gap between the general analysis of vaccine hesitancy delivered by the SAGE working group [10] and other systematic reviews that concentrate on one risk group and one flu type only [18–21]. Furthermore, it integrates data on children and chronically ill individuals. With this approach we aim to gain a better understanding of influenza vaccine hesitancy as a broader concept for all relevant risk groups and influenza types. To support this goal, we applied a comprehensive search strategy that is unprecedented in research about influenza vaccine hesitancy.

### Models of vaccine hesitancy

There are several levels at which we can analyze vaccine hesitancy. On the meso-level, the SAGE working group proposed in their model that individual/social influences, contextual influences and vaccine and vaccination-specific issues play a role [10,13]. It is further broken down and described as being “influenced by factors such as complacency, convenience and confidence” [10,13] as well as calculation on the macro-level [22]. High complacency implies that “…general involvement in the decision is low because complacent individuals do not feel threatened by infectious diseases” [22]. Low convenience (or inconvenience) can emerge because “attitudes are not strongly against or in favor of vaccination in this case, which means that vaccination is not important enough to actively overcome barriers. Consequently, when decision-makers face barriers such as lack of access, cost, or travel time, they decline vaccination to avoid these barriers” [22]. A lack of confidence usually emerges due to “strong negative attitudes towards vaccination (in contrast to the complacency and convenience types)” [22]. This in turn is correlated with the belief in misconceptions about the vaccine and the disease because knowledge “is likely to be distorted by misinformation about risks posed by vaccination or by affiliation to certain social groups close to the anti-vaccination movement” [22]. Calculation plays a role when “individuals do not have a strong pre-existing attitude towards vaccination but base their decisions on utility maximization, which leads to vaccination or non-vaccination, depending on the subjective evaluations of risks.” [22].

On the micro-level, the model can be further specified and characterized by psychological profiles that refer to psychological theories of health decision-making and behavior [22], such as the Theory of Planned Behavior (TPB; [23]). These models provide psychological insights that contribute to our understanding of why some individuals get vaccinated while others refuse to do so. They define the potential barriers to vaccine uptake on a concrete and measurable level, and specify the interrelation between the variables. TPB describes health behavior as a function of the behavioral intention to show a certain behavior (e.g. vaccination). The intention is a function of an individual’s attitude (negative or positive evaluation of behavior and outcome), perceived behavioral control (PBC; perceived ability to perform a behavior) and the subjective norm (perceived social pressure of significant others). Literature about social norms distinguishes between injunctive norms (i.e. what significant others think one should do) and descriptive norms (i.e. what significant others do). In the TPB, subjective norms are injunctive norms, defined as the product of normative beliefs (what others think one should do) and one’s motivation to comply with these beliefs. Based on a meta-analysis Revis and Sheeran suggest to also include descriptive norms in the TPB to increase its predictive power [24]. Other extensions of the TPB have also shown an increase in the predictive power of the theory by integrating concepts of risk perception [25], past behavior [26], knowledge [27], and experience [28] into the model. TPB thus reliably predicts various health behaviors, such as vaccinating, condom use, or physical activity [29].

**Fig 1:**
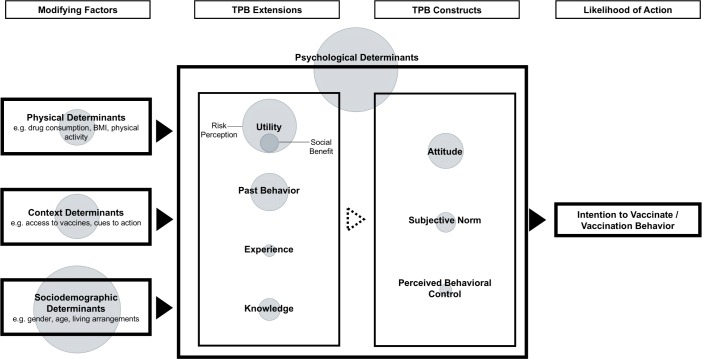
Micro-level determinants of vaccine hesitancy following an extended version of the Theory of Planned Behavior (TPB). Determinants are based on empirical and theoretical work from Ajzen [23], Schmiege et al. [25], Rhodes & Courneya [26], Koo et al. [27], Pomery et al. [28], Larson et al. [10]. Significant determinants from 470 research articles are clustered according to the model. Circle size represents the total number of reported incidents.

In this review we will analyze vaccine hesitancy on the macro- and micro-level. The results section reports the barriers to influenza vaccination on the micro-level for different risk groups and across influenza types. We use an extended version of TPB as a comprehensive theoretical framework for identifying and clustering barriers to influenza vaccination that have been identified during the past decade. As this review aims to integrate all potentially relevant barriers rather than psychological processes alone, we also include physical, contextual, and sociodemographic aspects to the conceptual framework [10,13]. This enhanced framework is displayed in Fig 1. In the discussion section we will integrate the findings on the macro-level and discuss hesitancy profiles of the different risk groups, also regarding potential differences of seasonal vs. pandemic influenza within these groups.

### Goals of the present review

With this framework the systematic review identified relevant studies on influenza vaccine hesitancy published between 2005–2016 to address the following research goals:

1. to extract individual barriers to seasonal and pandemic influenza vaccination for risk groups and the general public;
2. to map knowledge gaps in understanding influenza vaccine hesitancy and to derive directions for further research and interventions in this area.

A comprehensive understanding of influenza vaccine hesitancy in different risk groups and under different circumstances (seasonal vs. pandemic) is necessary to develop evidence-informed strategies and research to increase coverage of seasonal influenza vaccines. The review contributes to the 2016 consultation on the Global Action Plan for Influenza Vaccines (GAP). One of the objectives of the GAP is to increase the seasonal uptake of influenza vaccines to improve pandemic preparedness [30]. WHO has launched GAP in order to reduce the threat and burden of influenza for individuals and populations alike.

## Method

### Search and selection procedure

This review uses databases in the areas of Medicine, Bioscience, Psychology, Sociology and Public Health, in order to capture the great variety of aspects that define influenza vaccine hesitancy. The search was also extended to Global Index Medicus (GIM) libraries [31] that provide health literature produced by low- and middle-income countries. These libraries help to identify research from areas that are under-represented in research about vaccine hesitancy [10]. The final search included the following databases:

**Table:** 

• Medline	• LILACS
• Embase	• IBSS
• PsychInfo	• IMEMR (GIM)
• Cinahl	• IMSEAR (GIM)
• The Cochrane Library	• AIM (GIM)
• Web of Science	• WPRIM (GIM)

Table 1 shows the search terms used in this review. From these, a broad search string was first developed for PubMed (see S1 Table) and then adapted to all other databases. Guided by previous systematic reviews, we included terms in the review that relate to the broader concept of vaccine hesitancy ([10]; e.g. acceptance, demand, refusal), and specify more detailed psychological terms ([4,19,21,32]; e.g. attitude, knowledge, belief). Furthermore, terms that appear in a broader policy-oriented discussion of vaccine hesitancy were also included ([33]; e.g. policy, mandatory, trust).

**Table 1:** Keywords used for the literature search.

	AND		AND	knowledge	misconception*	misinformation	promoter*	barrier*
influenza*		vaccin*		behavior	delay	criticis*	controvers*	anxi*
seasonal influenza*		immuniz*		behavior	choice*	doubt*	oppos*	confidence
pandemic influenza*		Immunis*		uptake	hesitan*	exemption*	dilemma*	trust
H5N1		Inoculat*		intervention*	demand	rejection*	objector*	distrust
H1N1		prevention and control		increas*	accept*	rumor	determinant*	mistrust
flu				decreas*	refus*	rumour	attitude*	awareness
				dropout*	denial*	mandatory	belief*	fear*
				decision making	concern*	anti-vaccin*	emotion*	perception*
				campaign*	recommend*	polic*	intent*	compulsory

Note: Truncation was removed when a MeSH/CINAHL or EMTREE term matched the concept. When databases provided thesaurus services (e.g. Medline MeSH; Cinahl CINAHL; Embase EMTREE) keywords were exploded. This means that in these databases certain search terms possessed synonyms (e.g. in Medline the so-called MeSH-Terms for decision-making were Choice Behavior, Negotiating, Uncertainty, Consensus, Dissent and Disputes, Avoidance Learning Decision Making). Upon searching for “decision-making” all specific subterms were automatically entered in the search.

Terms regarding influenza and its prevention by vaccination were added to the resulting list of search terms to specify the search (columns 1 and 2 in Table 1). Thus, the search included results that were about influenza AND vaccines AND one of the hesitancy indicators specified in the remaining five columns of Table 1. The publication dates of interest were limited to the period between 01.01.2005 and 18.01.2016.

**Fig 2:**
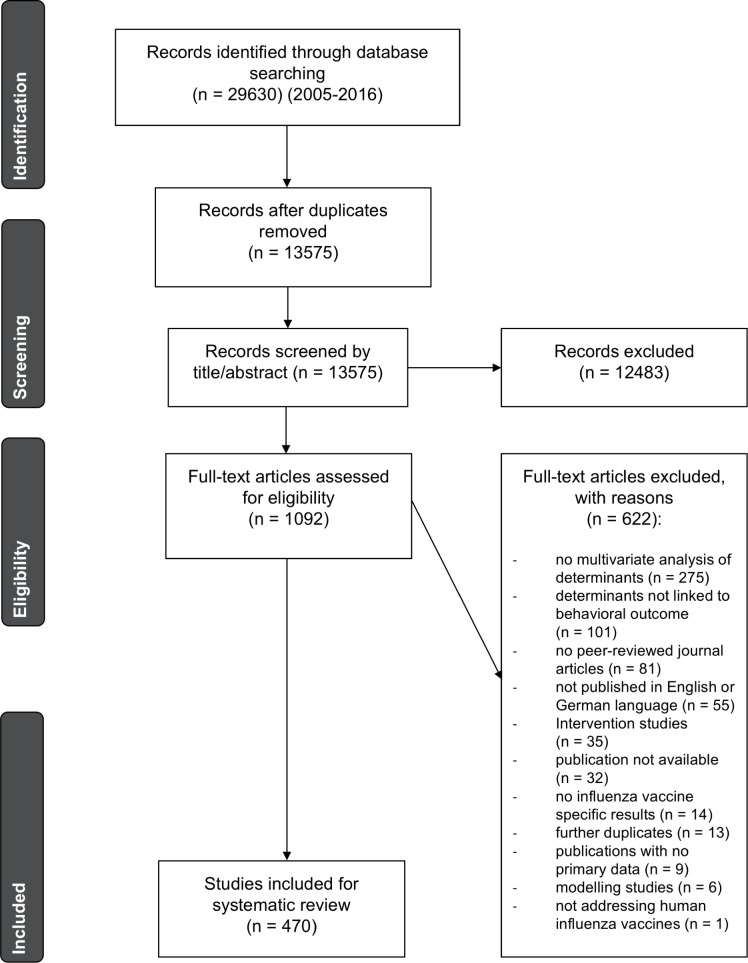
Flow Chart of selection process.

The initial search was conducted from 18.01.2016 to 12.02.2016. The analysis followed the PRISMA-approach [34] displayed in Fig 2. After duplicates were removed, all remaining articles were first scanned by title and abstract. Then, articles were excluded according to the a priori exclusion criteria which are displayed in Table 2. Since the focus of the review was on barriers to human influenza vaccine uptake or intention, reasons for exclusion were: not addressing human influenza vaccine, content not related to determinants of influenza vaccine hesitancy (e.g. studies about effectiveness of the vaccine), determinants not linked to a behavioral outcome. To ensure quality of results articles that were not peer-reviewed were excluded. To avoid the risk of including the same study twice and to avoid issues with conflicting exclusion criteria, other review articles and meta-analysis with no primary data were excluded. Because modeling studies and intervention studies were not the focus of this review, they were excluded. Due to language barriers, articles not in English or German were also excluded. Most databases provided filters for the exclusion criteria year of publication (2005–2016), species in focus (human) and type of publication (journal article). If applicable, these filters were used during the initial search.

**Table 2:** Exclusion criteria used in selection process for literature review on influenza vaccine hesitancy.

▪ not addressing human influenza vaccine
▪ content not related to determinants of influenza vaccine hesitancy
▪ determinants not linked to a behavioral outcome
▪ not a peer-reviewed journal article
▪ not reporting primary data (including other reviews and meta-analysis)
▪ modelling study
▪ intervention study
▪ not published in English or German language
▪ not published between 2005 and 2016
▪ not reporting multivariate analysis of determinants

The resulting articles were coded by year of publication, WHO region, risk group, influenza type, outcome variable (intention or behavior), and type of analysis (multivariate vs. other). The category *other* includes studies that used univariate analysis (models including only one independent predictor), correlative studies and qualitative studies. To acknowledge the multidimensional nature of vaccine decision-making and to increase validity of reported barriers, only articles using multivariate analysis were further analyzed (see also [10]). From the remaining studies, all significant predictors of influenza vaccine uptake or intention (p-value < .05) were extracted and documented.

### Coding and operationalization of vaccine hesitancy

Vaccine hesitancy is thus operationalized as low influenza vaccine uptake or low intentions to vaccinate against influenza. Therefore, each predictor is coded as a barrier, i.e. as a determinant that decreases uptake or intentions. When lower age, for example, is related to lower uptake or lower intentions, lower age is coded as a barrier. When publications reported that higher age was associated with higher uptake, i.e. when it was framed as a “promoter” of vaccine uptake, we re-coded this so that lower age again corresponds with lower vaccine uptake. Only when the opposite result was found, e.g. lower age was related to higher uptake in one study *and* with lower uptake in another study, we coded age both as a promoter and a barrier. In cases of a non-linear trend, the association between the determinant and the outcome were coded as inconclusive (e.g. when individuals with highest *and* lowest age had significantly lower uptake rates than middle-aged groups). Additionally, for each study it was coded whether the intention to vaccinate or actual vaccine uptake was assessed.

## Results

### Identified literature

Overall, 29,630 records were identified using the described search strategy (Table 1) in the relevant databases described above. After removing duplicates, 13,575 articles were further screened by title and abstract (Fig 2). 12,483 papers were removed according to the exclusion criteria (Table 2). 1,092 articles were eligible for the full-text analysis. After full-text analysis, 590 articles were removed (Fig 2 for specific reasons). If a full article could not be found in any database used in this review, then authors were contacted and asked for access. This procedure was not successful in 32 cases. The remaining 470 articles were considered for descriptive analysis and synthesis.

### Descriptive analysis of articles

Research about barriers to influenza vaccine uptake was conducted in all WHO regions. However, most of the research focused on Western samples (Americas 199/470; Europe 176/470; Western Pacific 75/470; Eastern Mediterranean 12/470; Africa 5/470; South Eastern Asia 5/470). The majority of research did not focus on a specific risk group but addressed the general public (191/470). The most studied risk group was healthcare personnel (includes healthcare and non-healthcare occupations; e.g. administrative staff), while children between 6 and 59 months of age were the least studied group (Health Care Personnel 117/470; Elderly 62/470; Chronic Condition 45/470; Pregnant Women 35/470; unspecified22/470; Children 18/470). This trend is consistent over time and region. The largest proportion of research focused on seasonal influenza (331/470; pandemic influenza 156/470; avian influenza 5/470; unspecified 3/470). Actual vaccine behavior was the main outcome variable in the majority of the studies (377/470), while the intention to vaccinate against influenza was assessed in 100 of the 470 studies (unspecified 4/470). When the outcome was not specific for only one flu type, region, risk group or outcome (e.g. paper focused on pandemic and seasonal influenza but results were merged), then the publication was coded as unspecified. When a publication matched more than one category within a variable (e.g. when a study measured uptake and intention), then the publication was included in all separate descriptive analyses (e.g. for intention and behavior).

**Fig 3:**
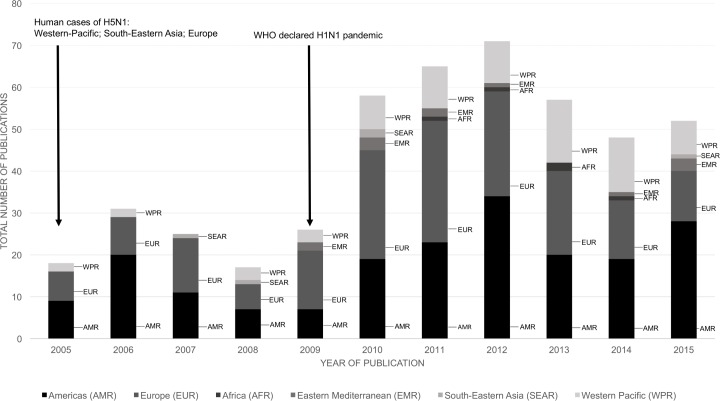
Total number of multivariate studies about influenza vaccine hesitancy by year of publication (2005–2015) and region (N = 470).

Fig 3 shows that over time the number of published studies about influenza vaccine hesitancy increased. There are peaks in the numbers of published articles after the avian influenza outbreak 2005, particularly in the Americas and European regions, and after the influenza pandemic 2008/2009 in the Americas, European and Western Pacific regions. The number of publications from authors from the African, South East Asian and Eastern Mediterranean regions remains low throughout the entire period.

### Micro-level analysis of determinants

After full-text analysis of all 470 articles, 258 independent barriers to influenza vaccination were identified. In order to identify the most relevant barriers, we counted the number of studies that found this barrier. The mean number of studies per barrier was M = 8.3, SD = 19.2. 104 barriers were significant in only one study. Due to the large quantity of identified variables, we introduced a cut-off criterion by including only those variables that were reported as significant barriers in at least 6 studies. This led to a total number of 72 barriers.

Fig 1 illustrates the number of significant results found in each category. The larger the size of the circles, the higher the number of significant results. The following micro-level analysis of influenza vaccine hesitancy provides information about the occurrence of each of the barriers in the different risk groups. The Supporting Information section additionally provides an illustration of all 72 predictors as increasing or decreasing factors of vaccination, stratified for each risk group and flu type (S1–S13 Figs). As the results for intention and uptake are highly similar, we have aggregated the results and refer to both outcomes as vaccine uptake.

### Psychological barriers

#### Utility

The utility of vaccination is a function of benefits and risks associated with vaccination (e.g. issues around vaccine safety). Benefits arise through protection from disease, i.e. the perception of disease risk. Additionally, vaccination entails a social benefit through herd immunity. In terms of measurement, research about risk perception differentiates between risk as analysis [35] vs. risk as feelings [36]. The former is measured as a combination of perceived severity of the disease or vaccine-adverse events (VAE) and the probability of getting the disease/VAE [37]. The latter is the result of emotional concern, which was previously operationalized as worry about risk of the disease/VAE and anticipated regret of (not) receiving the vaccine [38].

#### Risk perception

Perceiving low risk of the disease was identified as a barrier to influenza vaccine uptake in most risk groups and the general public (HCP 18/117 [39–56]; Pregnant 1/35 [57]; Chronic 1/45 [58]; Elderly 2/62 [59,60]; Public 12/191 [61–72]). For example, an Australian study found that among members of the general public the two most stated reasons for not accepting the vaccine were “situation is not serious enough” and “I am not at risk” [70]. In line with this, the cognitive parameters of risk perception were frequently identified as significant barriers, i.e. perceiving the likelihood of getting the disease as low (HCP 4/117 [43,73–75]; Pregnant 1/35 [76]; Elderly 1/62 [77]; Public 9/191 [68,72,78–84]) and perceiving the severity of the disease as low (HCP 8/117 [44,85–91]; Pregnant 3/35 [92–94]; Chronic 3/45 [58,95,96]; Children 2/18 [97,98]; Elderly 4/62 [96,99–101]; Public 16/191 [65,71,72,81,83,99,102–111]). Additionally, perceiving oneself as less susceptible to the disease decreased the uptake of the influenza vaccine in 23 studies (HCP 4/117 [42,112–114]; Pregnant 3/35 [115–118]; Chronic 1/45 [95]; Children 1/18 [119]; Elderly 1/62 [99]; Public 11/191 [99,107,108,120–128]).

Of all 470 studies, 42 reported affective parameters of risk perception as significant barriers to influenza vaccination, i.e. low worry about the disease (HCP 7/117 [39,40,129–133]; Pregnant 3/35 [117,134,135]; Elderly 1/62 [136]; Public 24/191 [61–64,79,102,120,121,137–152]) and low anticipated regret in the case of not being vaccinated (HCP 1/117 [153]; Pregnant 1/35 [134]; Public 5/191 [78,154–157]). For example, Tucker et al. report that pregnant women in a US sample who worried about influenza were approximately 3 times more likely to get the flu vaccine even when adjusting for cognitive risk parameters like probability and susceptibility of the disease [135].

Cognitive and affective risk perceptions regarding the vaccine were also reported as a barrier to influenza vaccination. Specifically, higher perceived risk of vaccine adverse events was found to decrease vaccine uptake (HCP 7/117 [41,158–163]; Pregnant 5/35 [94,164–167]; Chronic 2/45; Children 1/18 [98]; [58,96]; Elderly 3/62 [96,136,168]; Public 9/191 [66,83,109,111,169–173]), as was general worry about the safety of the vaccine (HCP 15/117 [44,45,53,56,75,87,90,112,130,132,159,163,174–176]; Pregnant 6/35 [92,94,117,167,177,178]; Elderly 1/62 [99]; Children 2/18 [179,180]; Public 14/191 [66,103,137–139,154,169,172,173,181–185]). For example, in an Australian study beliefs like, "1) the vaccine had been rushed through; 2) there had been insufficient research; 3) the vaccine had not been tested adequately" [70] were among the expressions of concern regarding the safety of the vaccine.

#### Social benefit

Of all 470 studies, 29 identified the social benefit as a significant influence on influenza vaccine uptake. The social benefit of vaccination is often used as an ethical argument for HCP to get vaccinated [186]. Thus, most studies in this section pertain to HCP. Individuals who did not acknowledge the social benefit of the vaccine were less likely to vaccinate (HCP 3/117 [86,89,187]; Public 3/191 [128,188,189]). When healthcare personnel lacked the belief that getting vaccinated protects patients (HCP 5/117 [45,53,161,187,190]) or relatives (HCP 2/117 [161,187]) vaccine uptake was lower; the same was found for pregnant women regarding their unborn child (1/35 [177]). Moreover, when individuals perceived low risk for others due to influenza, their uptake was lower (HCP 3/117 [86,89,187]; Public 6/191 [61,66,140,141,191,192]). The perception that there is low risk of transmitting the disease to others also decreased uptake (HCP 8/117 [43,44,46,55,75,158,175,193]). Variables referring to the social benefit of vaccination were not reported as a significant influence for the elderly, chronically ill patients and children under 5 years of age.

#### Subjective norm

When individuals perceived low pressure of significant others to get vaccinated, vaccine uptake was lower than when social pressure was high (HCP 4/117 [88,91,153,194]; Pregnant 1/35 [195]; Chronic 2/45 [196,197]; Public 10/191 [66,111,124,155,157,198–202]). In some studies the descriptive norm (the belief about what others do) was related to higher uptake (HCP 2/117 [203,204]; Pregnant 1/35 [134]). Yet other studies reported a lower normative influence, based on a score that includes injunctive norms, i.e. the belief in what others think one should do, and descriptive norms (Children 1/18 [205]; Public 5/191 [206–210]), as a barrier to vaccine uptake. For HCP, vaccine uptake was lower when they lacked the belief that the vaccine was an ethical or professional obligation (10/117 [48,194,211–218]). For example, a Dutch study found that HCPs had a lower likelihood of getting vaccinated when they did not acknowledge that it was their duty to do no harm and to ensure continuity of care [55]. In the general public, one study assessing subjective norms was inconclusive [219] and one experimental study found free-riding behavior in a hypothetical setting, i.e. declining vaccination uptake with an increasing number of vaccinated individuals [83].

#### Perceived behavioral control

6 articles found that lacking perceived behavioral control was a significant barrier to vaccine uptake (Public 5/191 [124,155,199,200,220]; Unspecified 1/22 [221]); as was low self-efficacy (HCP 1/117 [153]; Elderly 1/62 [222]; Children 1/18 [223]; Public 5/191 [124,209,210,224,225]). For example, a study by Hilyard et al. found that “parents were 1.3 times more likely than others to get their children vaccinated for every standard deviation increase in self-efficacy” [226].

#### Attitude

Having a negative attitude towards the influenza vaccine was a major barrier to vaccine uptake (HCP 7/117 [153,161,227–231]; Pregnant 6/35 [92,232–236]; Chronic 4/45 [197,237–239]; Elderly 1/62 [222]; Public 17/191 [12,65,124,128,143,155,198–200,202,220,240–245]). Moreover, individuals who did not believe in the effectiveness of the vaccine showed lower vaccine uptake in every risk group (HCP 11/117 [44,46,48,75,86,130,161,190,194,211,246]; Pregnant 5/35 [76,116,195,235,247]; Chronic 4/45 [58,96,248,249]; Children 1/18 [250]; Elderly 1/62 [96,99,100,136,251–253]; Public 23/191 [61,65–67,81,99,102,104,105,109,121,144,156,169,173,181,185,199,224,254–257]). In one study about maternal influenza immunization decision-making, Frew et al. note that “a woman who believed the vaccine was highly effective had a 30% to 60% increase of likelihood of later obtaining it” [195]. Additionally, a lack of trust in authorities such as the National Health Service (NHS) was reported to hinder immunization (1/117 HCP [87]; Pregnant 2/35 [117,167]; Elderly 1/62 [258]; Public 20/191 [63,81,101,108,120,122,141,142,147,172,173,191,198,201,259–264]).

#### Past behavior

Individuals who had already been vaccinated against influenza in previous seasons showed higher vaccine uptake in all risk groups (HCP 43/117 [42,44,50,51,54,74,75,86,87,112,113,160,161,190,193,214,216,230,265–289]; Chronic 9/45 [197,248,249,290–295]; Pregnant 10/35 [76,93,115,116,167,178,195,296–298]; Children 2/18 [223,299]; Elderly 7/62 [100,168,258,300–303]; Public 50/191 [62,66,69–72,81,99,101,103,125,140,143,145,146,150,152,157,169,170,172,173,183,191,192,198,209,219,224,240,254,256,259,303–320]). These findings mirror the results of previous systematic reviews that have repeatedly identified past behavior as a strong predictor of influenza vaccine acceptance [18,19,21]. Additionally, uptake of influenza vaccine was positively related to uptake of pneumococcal vaccination in chronically ill patients and the elderly (4/45 [290,291,295,321]; elderly (1/62 [322]).

#### Experience

9 studies related the experience of sickness to vaccine uptake: individuals who had not suffered from influenza previously were less likely to be vaccinated in upcoming seasons (HCP 4/117 [75,230,273,323]; Pregnant 1/35 [324]; Chronic 1/45 [325]; 1/18 Children [119]; Public 2/191 [326,327]). In one study, however, HCP who had been infected with H1N1 were less likely to get vaccinated [86]. This finding could be explained by the fact that participants believed they had already been infected with the very same strain the vaccine protects against. In the healthcare context, 5 studies noted fewer years of professional experience as a barrier to vaccine uptake in HCP (5/117 [194,285,328–330]). For example, Looijmans van den Akker et al. found lower vaccine uptake when HCPs had worked in healthcare for less than 15 years [194]; Dönmez et al. reported lower vaccine uptake for physicians who had worked for less than 5 years [330].

#### Knowledge

In all risk groups as well as the general public, lacking general knowledge about influenza and the vaccine was identified as a barrier (HCP 11/117 [41,51,53,131,277,285,328,331–334]; Pregnant 4/35 [93,233,236,335]; Chronic 2/45 [238,325]; Children 1/18 [336]; Elderly 1/62 [252]; Public 9/191 [110,314,326,337–342]). Examples of knowledge are modes of transmission of the disease and medical exemptions for vaccination. For HCP a lack of influenza-specific education, such as influenza-related training or education materials, was a barrier to vaccine uptake (5/117 [52,131,272,283,343]). The belief in misconceptions, i.e. agreement with specific false statements, was reported as a barrier for HCPs (4/117 [46,161,273,344]) pregnant women (2/35 [117,167]), chronically ill individuals (1/45 [248]), the elderly (1/62 [77]), and the general public (2/191 [220,255]). The majority of articles report the myth that the vaccine can cause the flu as a significant barrier. For pregnant women, misconceptions regarding the safety of the vaccine for the fetus, e.g. the increased chance of miscarriage or birth defects, were reported as a definitive barrier [167]. Additionally, one study reported an increase in vaccine uptake when individuals wrongly believed that the vaccine protects against the common cold [316].

### Physical barriers

Several studies have found that unhealthy lifestyles have a negative impact on vaccine uptake, such as alcohol consumption (Chronic 1/45 [321]; Public 3/191 [345–347]) and smoking habits (Pregnant 3/35 [296–298]; Chronic 11/45 [95,249,321,325,348–354]; Elderly 9/62 [346,350,355–361]; Public 9/191 [317,346,347,361–366]). However, smoking was also a promoter in some cases (HCP 1/117 [367]; Public 3/191 [72,192,368]), and alcohol consumption did not reveal a clear picture in the reviewed studies (Pregnant 1/35 [297]; Elderly 1/62 [346]; Public 2/191 [346,365]). More consistently, having given up smoking was reported to increase the uptake of vaccination (HCP 2/117 [367,369]; Chronic 4/45 [321,370–372]; Elderly 3/62 [346,361,373]; Public 6/191 [192,318,346,347,373,374];). The relationship between these rather proximate health variables and vaccine uptake “may be explained by confounding factors, such as health status, attitudes regarding immunization and physician’s perspective of smokers’ health, so this association should be interpreted with care” [353].

The results concerning the level of physical activity and one’s own perceived health status are similarly mixed. Decreased physical activity was reported as a barrier to vaccination in some cases (HCP 1/117 [289]; Elderly 2/62 [361,375]; Chronic 1/45 [372]; Public 4/ 191 [316,347,361,365]), and as a promoter in others (Chronic 1/45 [321]; Public 1/191 [360]). When individuals perceived their own health status as good they were less inclined to vaccinate (Chronic 5/45 [321,350–352,371]; Elderly 6/62 [350,355,361,376–378]; Public 7/191 [360,361,374,377,379–381]). This correlation, however, was reversed in a few other studies (Chronic 1/45 [382]; Elderly 2/62 [383,384]; Public 2/191 [338,363]).

Further consistent barriers to influenza vaccine uptake were a lower body mass index (Chronic 1/45 [321]; Elderly 2/62 [346,366]; Public 2/191 [346,366]) and not having a pre-existing medical condition (Chronic 5/45 [370,385–388]; Children 1/18 [389]; Elderly 3/62 [253,366,390]; Public 7/191 [137,169,253,257,264,319,380]).

### Contextual barriers

On the meso-level, the SAGE model acknowledges the influence of external contextual factors on vaccine uptake. We will differentiate between specific access issues (e.g. financial costs), interaction with healthcare system that can facilitate a vaccine decision [391] (e.g. having a regular source of care), cues to action as external triggers of vaccine behavior [392] (e.g. recommendation), and system factors that have been discussed as direct influences of healthcare utilization [391] (e.g. size of care facility can influence time of care per patient). All of these contextual factors describe potential external barriers that affect the ease of getting an influenza vaccine.

#### Access

General access to influenza vaccines due to political, geographical or economic issues influencing production and reliability of supply was not identified as a barrier to vaccination in this review. From the 470 studies reviewed, none reported a general lack of access to vaccines as a significant barrier to vaccine uptake. However, getting the vaccine was reported as inconvenient for HCPs (6/117 [42,45,56,86,88,393]), chronic patients (1/45 [196]), children (1/18 [205]) and the elderly (1/62 [99]). Matsui et al. note that “subjects described these barriers/inconveniences as means of transportation to a clinic, physical disability, and the expense of vaccination per se” [99]. Concrete financial expenses were reported as a barrier for HCPs (9/117 [91,112,160,215,273,285,343,394,395]), children (1/18 [205]) and the general public (7/191 [111,181,224,254,340,396,397]). For example, Kelly et al. found that among Australian students who were eligible for government-funded vaccines, the likelihood of getting the vaccine was 7 times higher than for individuals who were ineligible for funding [395].

#### Interaction with healthcare system

Individuals who interact less frequently with the healthcare system, e.g. fewer doctor visits [61] or hospitalizations in a given timeframe [95], had a lower likelihood of getting vaccinated (Pregnant 2/35 [324,398]; Chronic 12/45 [95,249,290,294,321,325,349,350,352,353,399,400]; Children 5/18 [119,401–404]; Elderly 21/62 [59,251,300,302,322,350,356–358,375,376,390,405–413]; Public 13/191 [61,255,304,347,405,414–421]). In line with these findings, not having a regular source of care (e.g. primary care physician) hindered vaccine uptake in most risk groups (HCP 2/117 [369,422]; Pregnant 1/35 [177]; Chronic 1/45 [370]; Elderly 3/62 [405,406,423]; Public 6/191 [192,264,363,368,405,424]).

#### Cues to action

Individuals who did not receive a direct recommendation from medical personnel were frequently reported to be less likely to vaccinate (HCP 5/117 [75,161,215,422,425]; Pregnant 15/35 [57,76,92,93,115,116,167,177,232–235,426–428]; Chronic 7/45 [58,248,291,292,429–431]; Children 6/18 [98,179,180,299,389,432]; Elderly 8/62 [96,100,136,222,300,322,407,433]; Public 11/191 [66,109,125,170,257,305,366,434–437]). The same was true for individuals who did not receive a recommendation from relatives (Elderly 3/62 [162–164]; Public 3/191 [103,125,219]).

#### System factors

The size of a healthcare facility (hospital, nursery home, practice) can impact vaccine uptake of patients, residents, or HCP employed in the facilities. 6 studies identified the size of the care facility as a significant factor for vaccine uptake. For HCPs, the results are rather inconclusive, with one study reporting increased size as a barrier (1/117 [343]) and 2 studies reporting it as a promoter (2/117 [438,439]). For patients and residents, an increased size of care facility was consistently noted as a barrier (Elderly 1/62 [302]; Public 2/191 [440,441]).

People who lived in the most socioeconomically deprived areas (1/45 Chronic [442]; 1/62 Elderly [442]; 2/191 Public [442,443]) or visited a practice in such areas (1/62 Elderly [444]) were identified as being less likely to receive influenza vaccination than people from wealthier areas.

### Sociodemographic barriers

Most sociodemographic factors present a mixed picture of results. As such, higher age was reported as a barrier to vaccine uptake (HCP 5/117 [53,54,89,285,331]; Chronic 3/45 [291,431,445], Children 3/18 [97,402,446]; Public 8/191 [72,108,224,309,396,447–449]) but also as a promotor (HCP 30/117 [45,46,51,55,75,91,131,133,274,280,289,323,374,450–465]; Pregnant 3/35 [57,297,324]; Chronic 21/45 [95,238,290,294,321,325,349–354,370–372,386–388,399,466–468]; Children 2/18 [119,205]; Elderly 21/62 [59,96,168,303,350,356,358,359,361,366,375,376,405,409,410,412,423,469–471]; Public 57/191 [61,63,68,69,71,83,120,122,142,145,147,155,172,241,255,264,303,306,307,312,313,316,318,341,347,360–363,366,368,374,380,381,384,397,401,405,415–418,435,441,471–483]). Moreover, some studies show inconclusive results regarding the influence of age (Chronic 1/45 [403]; Children 1/18 [484]; Elderly 2/62 [302,485]; Public 7/191 [105,148,152,307,311,419,486];). An even more inconclusive pattern can be observed for gender and ethnicity. Being female was noted as a barrier (HCP [50,51,53,133,279,283,289,425,451,453,458,487]; Chronic 7/45 [238,321,352–354,372,442]; Elderly 7/62 [59,301–303,322,346,485]; Public 21/191 [65,68,72,105,122,146,148,240,256,261,308,310,311,345,346,374,416,417,449,479,488]), as a promoter (HCP 10/117 [114,190,204,277,285,328,331,455,462,463]; Chronic 6/45 [96,249,370,466,489,490]; Elderly [96,366,423,491,492]; Public 19/191 [61,63,150,192,306,318,347,361,363,364,366,381,384,405,414,443,473,482,493]), and as inconclusive (Chronic 1/45 [403]; Public 2/191 [303,486]). Being white was reported as a barrier (Public 3/191 [70,137,494]) and somewhat more frequently as a promoter (HCP 3/117 [333,465,495]; Chronic 1/45 [370]; Children 1/18 [484]; Elderly 7/62 [361,405,409,469,496–498]; Public 7/191 [147,189,361,405,414,477,499]). The reasons why these variables either decrease or increase vaccine acceptance are rarely explained.

Living alone (Elderly 5/62 [59,168,375,470,500]) and being unmarried (Elderly 5/62 [350,355,360,361,469]; Public 5/191 [62,150,192,365,384,501]) was negatively associated with vaccine uptake. Nagata et al. (2013) mention that this relation may be mediated by access and cues to action: “people who live alone with limited assistance may have less access, irregular preventive health visits, and less support from family members” [18]. A limited number of other studies, however, found an inverse relationship of marital status and vaccination (Pregnant 1/35 [116]; Public 1/191 [150]). The results of the latter study by Frew et al. (2014) indicate that pregnant women who are single, divorced, or widowed “may exert more control over their health and the health of the unborn child, as they are likely the primary providers for themselves and their unborn child” and thus vaccinate more [116].

## Discussion

**Fig 4:**
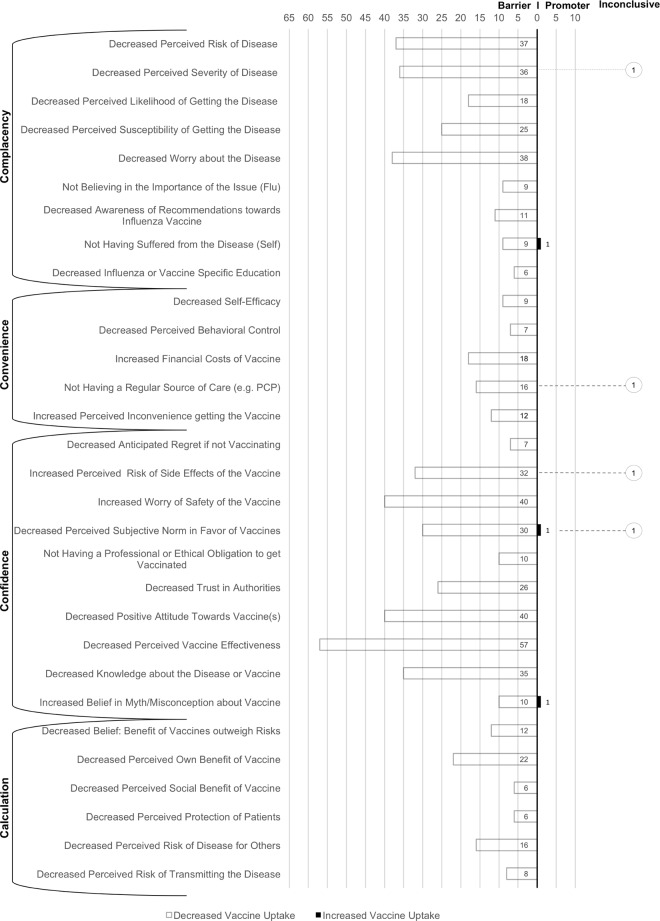
Barriers to influenza vaccine uptake mapped onto the macro-level 4C model of vaccine hesitancy (complacency, convenience, confidence and calculation). Total numbers of studies reporting the variable as either decreasing (white) or increasing (black) vaccine acceptance or inconclusive (circled number).

**Fig 5:**
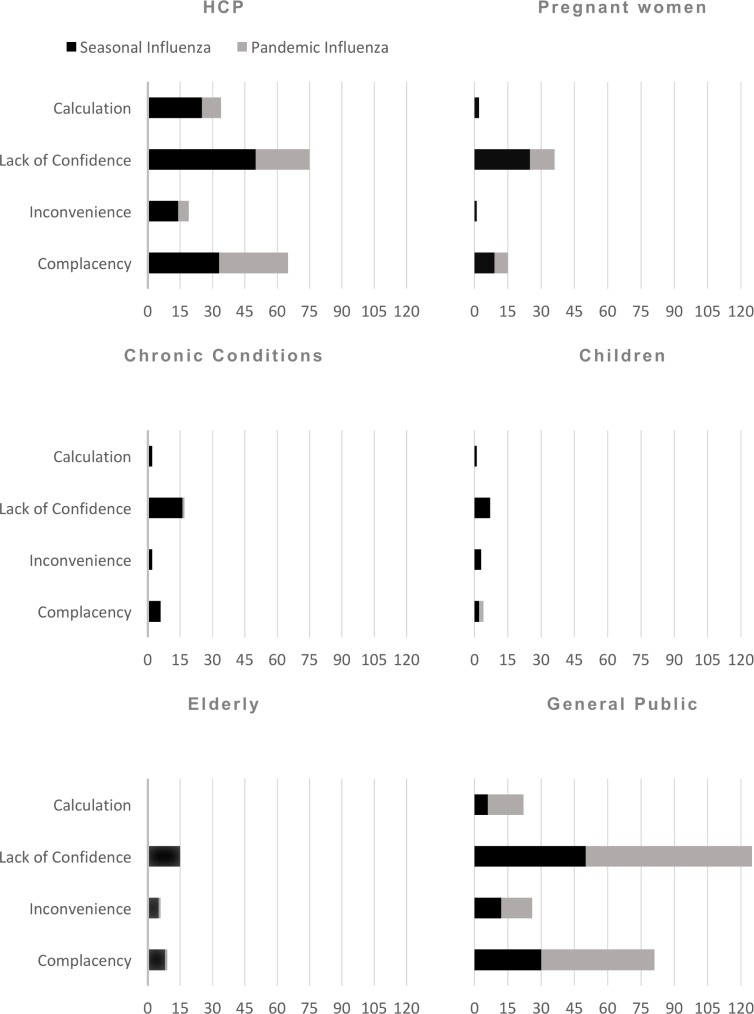
Barriers to seasonal and pandemic influenza vaccine uptake for each risk group aggregated using the macro-level 4C model of vaccine hesitancy (complacency, convenience, confidence and calculation). Bars visualize the total number of significant results supporting each reason for hesitancy for each risk group. Grey bars indicate the absolute proportion of results addressing pandemic influenza uptake among the total results. Black bars indicate the absolute proportion of results for seasonal influenza uptake among the total results.

On the micro-level, sociodemographic variables such as gender and age were the most reported, but also most inconsistent predictors of influenza vaccination. Besides this, lacking cues to action, low perceived utility of vaccination, a negative attitude towards influenza vaccines, and fewer previous influenza vaccinations were most frequently and consistently identified as significant barriers to influenza vaccination. In the following discussion we will first discuss differences between pandemic and influenza vaccine hesitancy and then summarize the evidence for each risk group stratified for pandemic and seasonal influenza vaccination. In doing this, we will first summarize the most important micro-level determinants and then synthesize the results based on the macro-level model of vaccine hesitancy. The macro-level synthesis intends to inform the design of future interventions to increase influenza vaccine uptake and thus supports our second research goal [22]. Fig 4 gives a detailed overview of which barriers we allocated to which of the four hesitancy reasons, i.e. lack of confidence (e.g. negative attitude towards vaccines, decreased trust in authorities), complacency (e.g. decreased perceived risk of the disease, decreased worry about the disease), calculation (e.g. decreased belief: benefit of vaccines outweighs risks), and inconvenience (increased financial costs of vaccine, decreased frequency of interaction with healthcare service). Fig 4 also provides an overview of the findings collapsed across risk groups and influenza types. Fig 5 visualizes the number of significant results supporting each reason for hesitancy for each risk group and for pandemic (grey bars) and influenza vaccination (black bars).

### Seasonal vs. pandemic influenza vaccine hesitancy across all risk groups

Barriers for seasonal and pandemic influenza uptake were very similar in kind and reported relative frequency. For both influenza types, having missed the influenza vaccine in previous seasons was the most frequently reported barrier to vaccine uptake. Likewise, the relative number of reports about the influence of age and gender and additional risk factors (e.g. being elderly and having a chronic disease) were similar for pandemic and seasonal influenza.

However, differences occurred with regard to the 4C profiles. From the perspective of the 4C model, studies about seasonal influenza revealed a lack of confidence as the most frequently reported barrier of vaccine uptake (e.g. negative attitude, misconceptions about the disease or the vaccine). For pandemic influenza, complacency was the major barrier to vaccine uptake (e.g. low worry and perceived risk of the disease), followed by confidence (increased worry about the safety of the vaccine; distrust in authorities). In times of crisis, laypersons have few options other than to base their decisions on predictions provided by health authorities, i.e. trust and risk perceptions are highly influential determinants. A lack of confidence due to low perceived effectiveness of the vaccine was frequently reported as a barrier to vaccine uptake for both flu types.

### Seasonal vs. pandemic Influenza vaccine hesitancy in HCP

For HCP, sociodemographic variables (age, gender, additional risk factors) and past behavior were among the most reported influences of seasonal as well as pandemic vaccine uptake. The profiles for pandemic and seasonal influenza vaccine hesitancy looked very similar (cf. S3 Fig and S4 Fig) with the exception that HCP who were not a physician showed lower uptake of the pandemic influenza vaccine.

On the macro-level, a lack of confidence was the major barrier to seasonal influenza vaccine uptake (due to decreased professional or ethical obligation to get vaccinated and misconceptions about the disease or vaccine). Additionally, complacency issues (decreased perceived risk of the disease) were also frequently reported. Inconvenience was the least reported reason.

For pandemic influenza vaccine uptake, complacency (decreased perceived risk of disease, decreased severity of disease, decreased worry about the disease) and lack of confidence (increased worry of safety of the vaccine) were similarly reported as prominent reasons for vaccine hesitancy in HCP.

### Seasonal vs. pandemic influenza vaccine hesitancy in pregnant women

Past behavior, higher age, and recommendation from medical personnel were the most frequently reported predictors for seasonal influenza vaccine uptake. A lack of confidence was the most frequently reported barrier to seasonal influenza vaccine uptake for pregnant women (high perceived risk of the vaccine, high worry about safety of the vaccine, low perceived effectiveness of the vaccine and misconceptions about the disease or vaccine).

For pandemic influenza, not having received a recommendation from medical personnel was the most reported barrier to vaccine uptake. Similar to seasonal influenza vaccine hesitancy, a lack of confidence was the most frequently reported barrier to pandemic influenza vaccine uptake among pregnant women (negative attitude, high perceived risk of the vaccine, high worry of safety of the vaccine and low perceived effectiveness of the vaccine).

### Seasonal vs. pandemic influenza vaccine in patients with chronic conditions

Previous behavior, smoking status and lower age were the most frequently reported barriers to seasonal influenza vaccine uptake among chronically ill patients. A lack of confidence due to a negative attitude and low perceived vaccine effectiveness and complacency issues (low perceived severity of disease) were the most reported barriers within the 4C framework for seasonal influenza.

For pandemic influenza, past behavior and higher age were also the most reported influences on vaccine uptake. Due to the low number of publications for chronically ill patients, on the macro-level only a lack of confidence due to misconceptions regarding the disease or vaccine were identified.

### Seasonal and pandemic influenza vaccine hesitancy in children

Sociodemographic variables (age and education) and missing recommendations from medical personnel for the decision maker, i.e. parents of children between 6–59 months of age, were the most reported barriers to seasonal influenza vaccine uptake for children. With regard to the 4C model, calculation (decreased perceived own benefit of vaccine) and inconvenience (decreased frequency of interaction with health service) were the most prominent reasons for vaccine hesitancy.

For pandemic influenza, the only reported barriers in this under-researched risk group were complacency issues (decreased perceived severity of disease, not believing in the importance of the issue).

### Seasonal vs. pandemic influenza vaccine hesitancy in the elderly

Sociodemographic variables (age, additional risk factors, education, gender), physical variables (smoking status, perceived health status) and past behavior were among the most reported barriers to seasonal influenza vaccine uptake. Especially for this risk group and in line with previous reviews [18], living arrangements (living alone and not being married) were reported as barriers to vaccine uptake.

On the macro-level, a lack of confidence (low perceived vaccine effectiveness, higher perceived risk of the vaccine) and complacency issues (lower perceived severity of disease, lower perceived risk of disease) were the most frequently reported 4C components among the elderly.

Studies about pandemic influenza vaccine hesitancy among the elderly were scarce. Complacency (low perceived severity of disease) and a lack of confidence (distrust in authorities) were the only reported barriers to vaccine uptake with regard to the 4C model.

### Seasonal vs. pandemic influenza vaccine hesitancy in the general public

For the general public, the uptake of seasonal influenza vaccine was most frequently related to sociodemographic variables (age, gender, education, income, additional risk factors) and past behavior. With regard to the 4C model, a lack of confidence (decreased perceived vaccine effectiveness, increased negative attitude towards vaccines and increased perceived risk of side effects of the vaccine) and complacency issues (decreased worry about the disease, decreased perceived severity of the disease) were most frequently noted as significant barriers to seasonal influenza uptake.

Pandemic influenza vaccine hesitancy was also most frequently related to sociodemographic variables (age, gender, education, additional risk factors) and past behavior. In line with the analysis across all risk groups, the most frequently reported barriers to pandemic influenza vaccine uptake for the general public were complacency (decreased worry about the disease, decreased perceived risk of the disease, decreased severity of the disease and decreased susceptibility to getting the disease) and a lack of confidence (decreased trust in authorities, decreased perceived vaccine effectiveness, decreased perceived subjective norm, increased worry about the safety of the vaccine and increased negative attitude towards the vaccine).

### The 4C vaccine hesitancy model

Across risk groups, all reasons against vaccination stated by the 4C model were identified as significant barriers of influenza vaccine hesitancy. However, convenience and calculation played only a minor role. The most frequently reported reasons for pandemic influenza vaccine hesitancy were complacency, especially due to low perceived risk and worry about the disease, and a lack of confidence due to distrust of authorities and decreased perceived safety of the vaccine. For seasonal influenza vaccination, a lack of confidence due to misconceptions and a negative attitude towards the vaccine was the most reported reason for seasonal influenza vaccine hesitancy. For both flu types and across all risk groups, a lack of confidence due to low perceived vaccine effectiveness was frequently reported.

The differences between the two influenza types with regard to their psychological profile of hesitancy as well as the differences between the risk groups described above can illustrate the benefits of applying the 4C model to intervention design. The model provides a heuristic for selecting and designing appropriate strategies to address and overcome vaccine hesitancy [22]. For example, if one aims to increase influenza vaccine uptake within a hospital setting, the results of this review suggest that addressing confidence (by debunking misconceptions and increasing awareness of an ethical and professional obligation to vaccinate) is a promising lever for interventions. Betsch et al. suggest that informational interventions such as educational campaigns are a suitable means by which to address low confidence. It is also shown that structural interventions such as mandatory vaccinations that are suitable to overcome complacency should be treated with care, as negative attitudes towards vaccination are a major barrier and could lead to reactance following structural interventions [22]. Combining the results from this systematic review with conceptual frameworks like the 4C model provides valuable insights about changeable barriers and the potentially effective strategies to overcome them.

### The predictive power of psychological variables

The majority of studies report sociodemographic variables as significant determinants of influenza vaccine hesitancy. However, it is important to note that most sociodemographic variables make little contribution to explain influenza vaccine hesitancy on an individual level. Firstly, inconclusive or contradictory findings were obtained especially frequently in the section of sociodemographic variables. Moreover, sociodemographic variables are at best a conglomeration of possible reasons and can never explain a specific behavior without further investigation. For example, a number of studies report an association between the ethnicity of a study population and influenza vaccine uptake [264,495,502,503]). Possible reasons for these findings might be differences in access to care [504], provider discrimination [505], negative attitudes towards vaccination [506], or trust in health authorities [507] among others [60]. Therefore, variables like ethnicity or gender are carrier variables [508] of explanatory factors rather than explanatory factors of hesitancy themselves. This means that these variables are potential confounders of variables that actually determine vaccine hesitancy. While such variables may be significantly related to vaccine hesitancy, they cannot be used to explain its emergence or intensity. Most importantly, without looking at psychological factors they are useless to inform interventions to counter hesitancy. While they may have some value in determining the target group of interventions, psychological variables should be used to inform the design of the intervention.

### Limitations

This systematic review describes the landscape of influenza vaccine hesitancy and its determinants rather than their weighted relative importance. Conclusions about the relative importance of determinants cannot be made. When a determinant is reported more frequently than another, then this can mean two things: (1) the variable is a better predictor of vaccine behavior or intention and turned out to be significant in more studies, or (2) the variable was studied more often. Variables like age and gender are the most frequently reported determinants. This probably does not mean that these are the most important variables, but rather that sociodemographic variables are always assessed as part of the research routine and therefore turn out to be significant in some studies. It is important to note that this review does not report and does not take into account studies that assessed the respective barriers and did not obtain significant associations. This was due to the high total number of studies obtained and the scope of the study. For the evaluation of the aggregated effect sizes of potential barriers and their relative importance, a meta-analytic approach is necessary. However, because measured barriers are rarely based on theoretical models and operationalization of constructs varies greatly between studies, meta-analytic approaches face tremendous difficulties addressing influenza vaccine hesitancy.

The majority of studies addressing influenza vaccine hesitancy were conducted in the American and European regions. Studies from all other regions are under-represented. Even though the number of studies for all risk groups has increased over the years, the proportion of studies for children, chronically ill patients and pregnant women remains relatively low. Thus, due to the limited availability of data, the conclusions of this review need to be limited to the regions and populations available. Additionally, the restriction of the search strategy to articles in German or English language may bias search results because it excludes articles from journals in other languages. The inclusion of Global Index Medicus (GIM) libraries may have reduced this bias.

### Directions for future research

As a response to the limitations previously discussed, future research needs to address the under-represented regions and populations. This way, designing interventions in all WHO regions and for all risk groups of influenza can base on evidence of influenza vaccine hesitancy.

It is not only important to improve who and which region is studied but also *what* is studied. Psychological variables provide insights to gain a better understanding of why some individuals refuse to get vaccinated while others do not. Our review may lead to the impression that psychological variables are frequently analyzed. However, the actual measurements are rarely based on psychological theories, and the items used to measure the constructs vary substantially across studies. For example, Loubet et al. measure knowledge by self-evaluation, while Tong et al. developed a 14 item knowledge-scale where the correctness of answers was used to calculate an individual knowledge score [233]. Moreover, especially constructs of risk perception (individual susceptibility, perceived probability, severity and overall judgments of risk) are rarely differentiated and used interchangeably across and even within publications. To ensure valid results and enable the scientific community to compare results between publications, we argue for the use of theory-informed scales of psychological determinants. This will improve the quality of future studies and ensure scientific progress within the research field of influenza vaccine hesitancy. For operationalization of constructs of the TPB and its extensions we provide a collection of items (see S2 Table). We encourage researchers of all disciplines to consider these measurements for future research on influenza vaccine hesitancy.

### Conclusion

The first goal of this review was to extract individual barriers to seasonal and pandemic influenza vaccination for risk groups and the general public. On a micro-level we used an extended version of the Theory of Planned Behavior. This review frames the determinants as barriers to vaccine uptake. With regard to the original model of the TPB, a negative attitude towards vaccines and attitudinal beliefs such as a decreased perceived effectiveness of the vaccine and a lack of trust in health authorities were the most frequently reported barriers. Perceiving that influenza vaccination was not the norm in the relevant peer group was frequently reported as a barrier. Relatively few research articles identified low perceived behavioral control as a significant barrier to influenza vaccine uptake. This might be due to the fact that perceived behavioral control is correlated with actual access. Research articles from regions with low reliability of vaccine supply were underrepresented (such as Africa, see Fig 3). This could lead to the overall impression that perceived or actual behavioral control does not play a role. Taking the extensions of the TPB into account, frequently reported additional barriers of influenza vaccine uptake were utility evaluations such as worries about the safety of the vaccine, low perceived severity of the disease and a low perceived risk of the disease. Furthermore, the lacking recommendations from medical personnel, low frequency of interaction with health services and missing vaccination habits were frequently reported as barriers. To frame it differently, the major and most consistent enablers for vaccine uptake on the micro-level were a positive attitude towards influenza vaccines, high perceived utility of vaccination, cues to action, and previous influenza vaccinations.

On a macro-level, the available evidence suggests that confidence, as well as complacency, are major reasons for influenza vaccine hesitancy. Complacency was mostly expressed by low worry, low perceived risk and severity of the disease. The lack of confidence was expressed by doubts about the safety and effectiveness of the vaccine as well as a lack of trust in health authorities and, among other knowledge gaps, the belief that the vaccine can cause the flu.

The second goal was to map knowledge gaps in understanding influenza vaccine hesitancy and to derive directions for further research in this area. We conclude that a more theory-based approach to measuring vaccine hesitancy–not only regarding influenza vaccines–will improve the knowledge base to design effective evidence-informed interventions.

By using evidence-informed approaches to measure and overcome vaccine hesitancy, the effectiveness of vaccine advocacy may be improved and the burden of vaccine-preventable diseases may be reduced.

## Supporting Information

S1 Table User query of relevant keywords in Medline via PubMed. (PDF)

S1 Fig Identified barriers to influenza vaccine uptake for all risk groups across seasonal and pandemic influenza. The figure visualizes the total numbers of studies reporting the variable as either decreasing (white) or increasing (black) vaccine acceptance or inconclusive (circled number).
(TIFF)

S2 Fig Identified barriers to seasonal influenza vaccine uptake for HCP. The figure visualizes the total numbers of studies reporting the variable as either decreasing (white) or increasing (black) vaccine acceptance or inconclusive (circled number).
(TIFF)

S3 Fig Identified barriers to pandemic influenza vaccine uptake for HCP. The figure visualizes the total numbers of studies reporting the variable as either decreasing (white) or increasing (black) vaccine acceptance or inconclusive (circled number).
(TIFF)

S4 Fig Identified barriers to seasonal influenza vaccine uptake for pregnant women. The figure visualizes the total numbers of studies reporting the variable as either decreasing (white) or increasing (black) vaccine acceptance or inconclusive (circled number).
(TIFF)

S5 Fig Identified barriers to pandemic influenza vaccine uptake for pregnant women. The figure visualizes the total numbers of studies reporting the variable as either decreasing (white) or increasing (black) vaccine acceptance or inconclusive (circled number).
(TIFF)

S6 Fig Identified barriers to seasonal influenza vaccine uptake for patients with chronic conditions. The figure visualizes the total numbers of studies reporting the variable as either decreasing (white) or increasing (black) vaccine acceptance or inconclusive (circled number).
(TIFF)

S7 Fig Identified barriers to pandemic influenza vaccine uptake for patients with chronic conditions. The figure visualizes the total numbers of studies reporting the variable as either decreasing (white) or increasing (black) vaccine acceptance or inconclusive (circled number).
(TIFF)

S8 Fig Identified barriers to seasonal influenza vaccine uptake for children aged 6 through 59 months. The figure visualizes the total numbers of studies reporting the variable as either decreasing (white) or increasing (black) vaccine acceptance or inconclusive (circled number).
(TIFF)

S9 Fig Identified barriers to pandemic influenza vaccine uptake for children aged 6 through 59 months. The figure visualizes the total numbers of studies reporting the variable as either decreasing (white) or increasing (black) vaccine acceptance or inconclusive (circled number).
(TIFF)

S10 Fig Identified barriers to seasonal influenza vaccine uptake for the elderly. The figure visualizes the total numbers of studies reporting the variable as either decreasing (white) or increasing (black) vaccine acceptance or inconclusive (circled number).
(TIFF)

S11 Fig Identified barriers to pandemic influenza vaccine uptake for the elderly. The figure visualizes the total numbers of studies reporting the variable as either decreasing (white) or increasing (black) vaccine acceptance or inconclusive (circled number).
(TIFF)

S12 Fig Identified barriers to seasonal influenza vaccine hesitancy for the general public. The figure visualizes the total numbers of studies reporting the variable as either decreasing (white) or increasing (black) vaccine acceptance or inconclusive (circled number).
(TIFF)

S13 Fig Identified barriers to pandemic influenza vaccine uptake for the general public. The figure visualizes the total numbers of studies reporting the variable as either decreasing (white) or increasing (black) vaccine acceptance or inconclusive (circled number).
(TIFF)

S2 Table Measurement of constructs of the Theory of Planned Behavior (TPB) and its extensions. (PDF)

S3 Table PRISMA Checklist. (PDF)
