# Supplementary material for: Barriers of Influenza Vaccination Intention and Behavior – A Systematic Review of Influenza Vaccine Hesitancy, 2005 – 2016
Source: PLoS One. 2017 Jan 26;12(1):e0170550. doi: 10.1371/journal.pone.0170550 (PMC5268454; doi:10.1371/journal.pone.0170550)
Supplement: S1 Table — (PDF) [file pone.0170550.s001.pdf]

User query of relevant keywords in Medline via PubMed: (Influenza vaccines OR ((influenza OR seasonal influenza OR pandemic influenza OR H5N1 OR H1N1 OR flu) AND (vaccines OR (prevention and control) OR immunization OR inoculation))) AND (behavior OR intention OR uptake OR intervention OR increase OR decrease OR dropout OR decision making OR delay OR choice OR hesitanc* OR demand OR accept* OR refus* OR denial OR concern* OR criticis* OR doubt* OR exemption* OR rejection OR rumor OR compulsory OR mandatory OR anti-vaccin* OR controvers* OR opposition OR dilemma* OR objector* OR determinant* OR attitude OR belief* OR emotion OR anxiety OR confidence OR trust OR distrust OR mistrust OR awareness OR fear OR perception OR knowledge OR misconception* OR misinformation OR barrier* OR promoter*)
