# Supplementary figures and images for: Barriers of Influenza Vaccination Intention and Behavior – A Systematic Review of Influenza Vaccine Hesitancy, 2005 – 2016
Source: PLoS One. 2017 Jan 26;12(1):e0170550. doi: 10.1371/journal.pone.0170550 (PMC5268454; doi:10.1371/journal.pone.0170550)

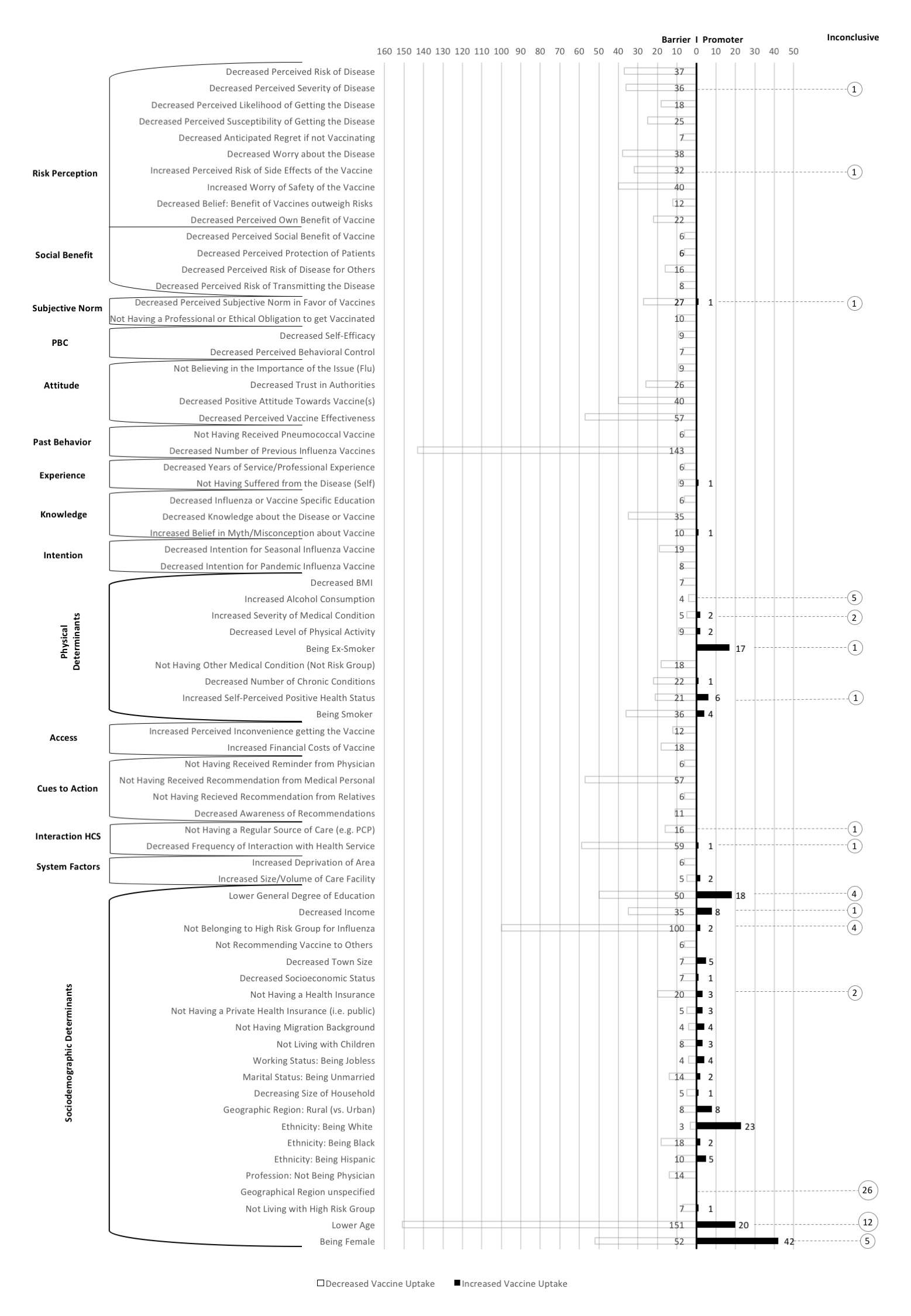

Supplement: S1 Fig — The figure visualizes the total numbers of studies reporting the variable as either decreasing (white) or increasing (black) vaccine acceptance or inconclusive (circled number). (TIFF) [file pone.0170550.s002.tiff]

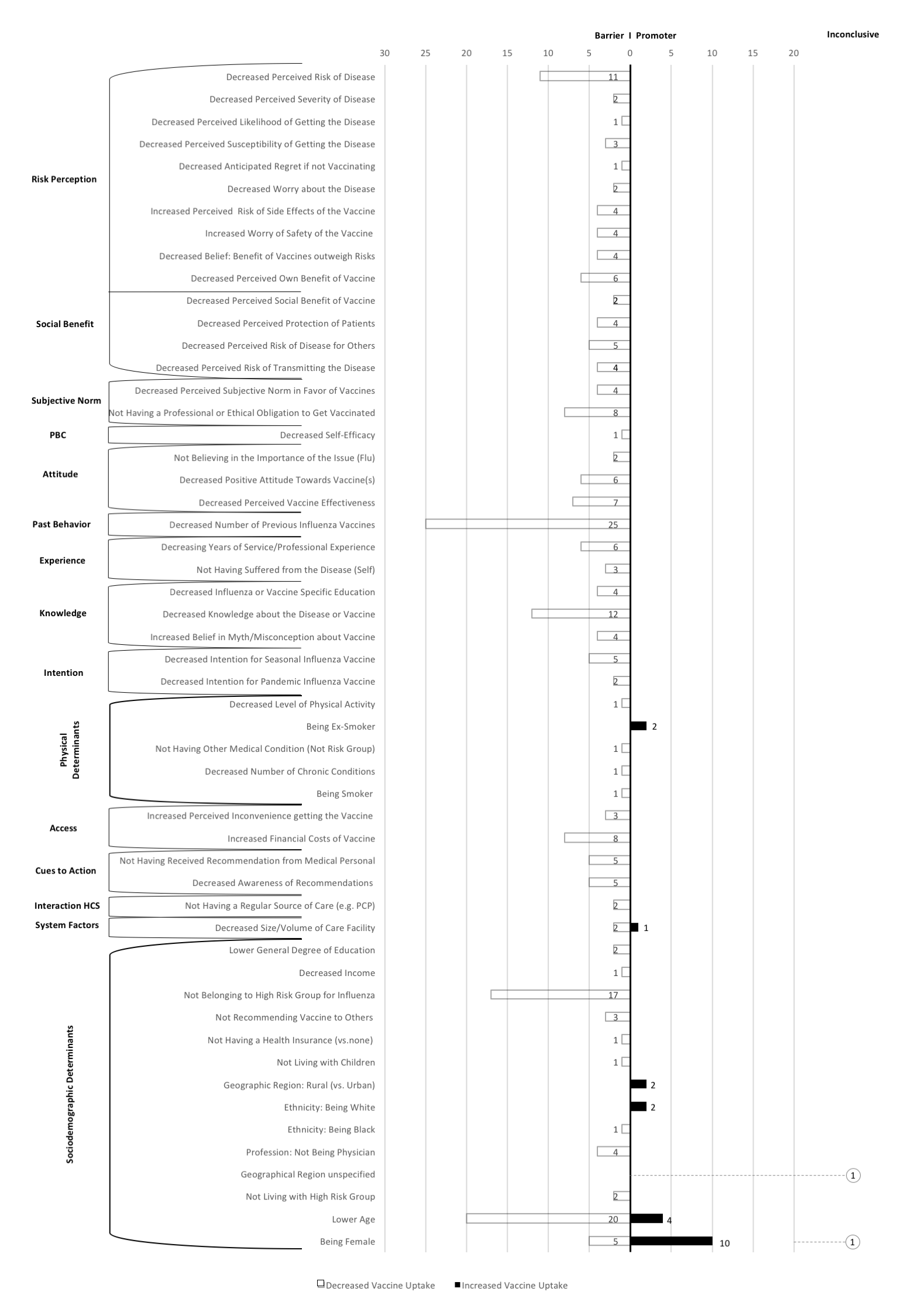

Supplement: S2 Fig — The figure visualizes the total numbers of studies reporting the variable as either decreasing (white) or increasing (black) vaccine acceptance or inconclusive (circled number). (TIFF) [file pone.0170550.s003.tiff]

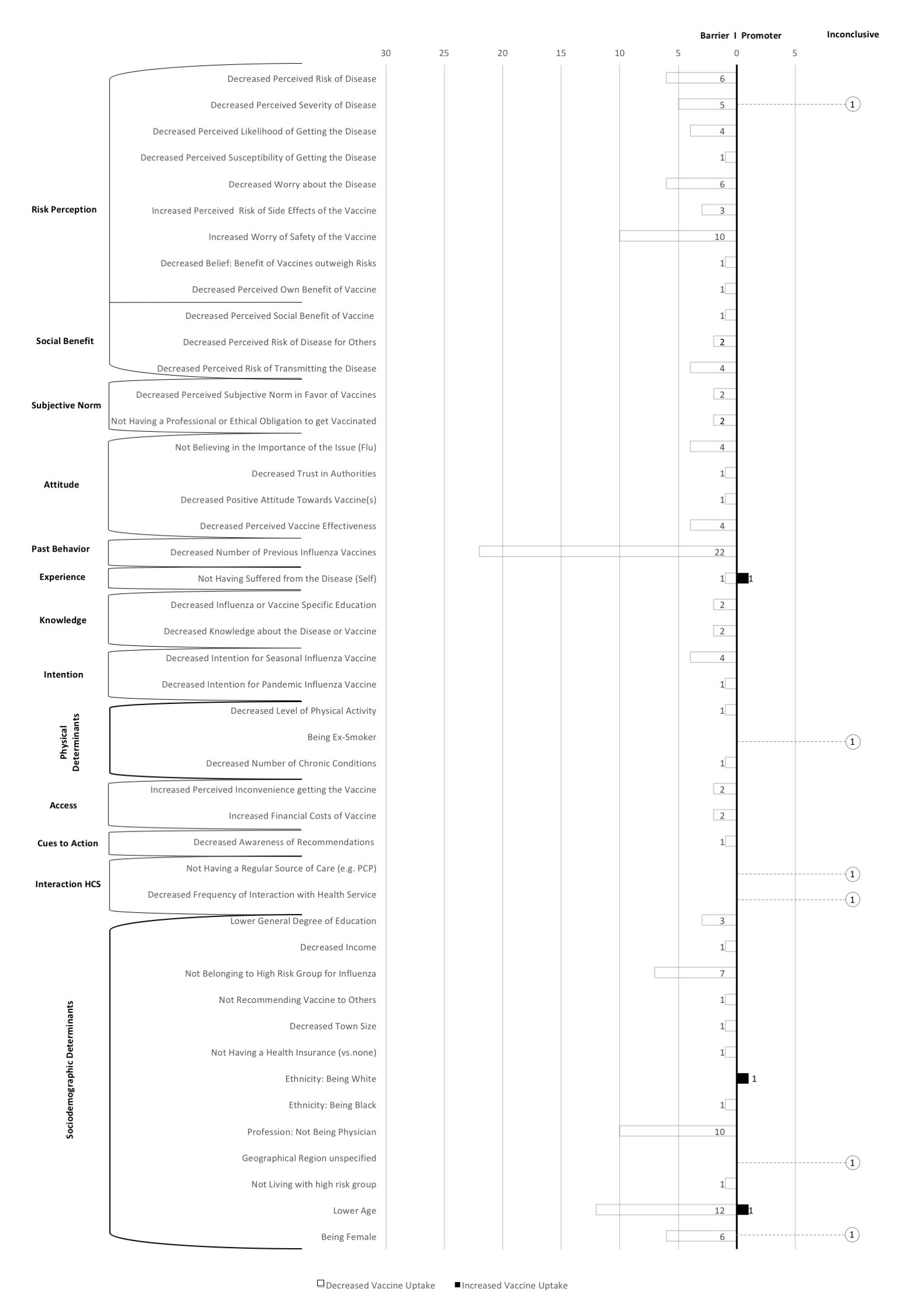

Supplement: S3 Fig — The figure visualizes the total numbers of studies reporting the variable as either decreasing (white) or increasing (black) vaccine acceptance or inconclusive (circled number). (TIFF) [file pone.0170550.s004.tiff]

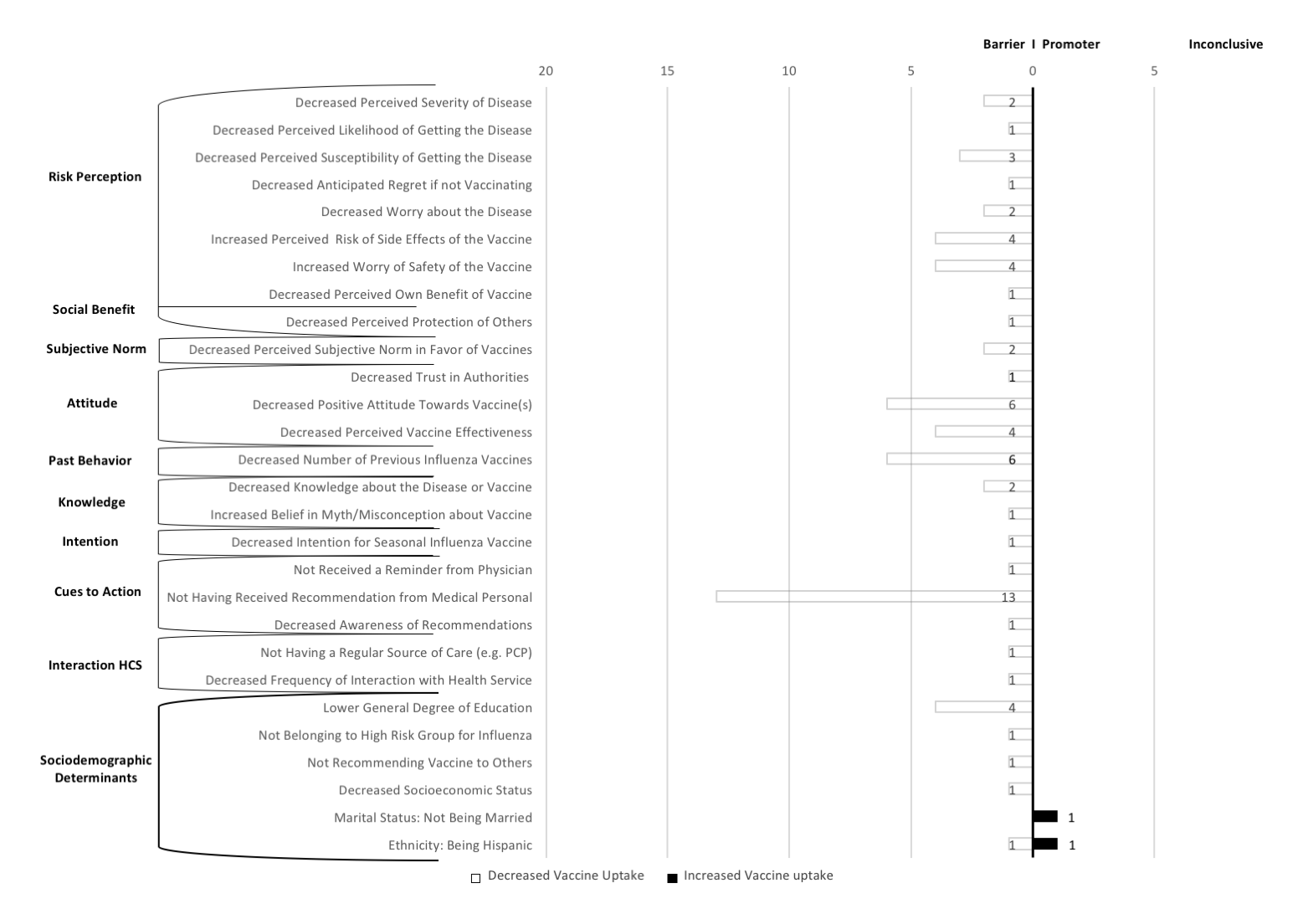

Supplement: S4 Fig — The figure visualizes the total numbers of studies reporting the variable as either decreasing (white) or increasing (black) vaccine acceptance or inconclusive (circled number). (TIFF) [file pone.0170550.s005.tiff]

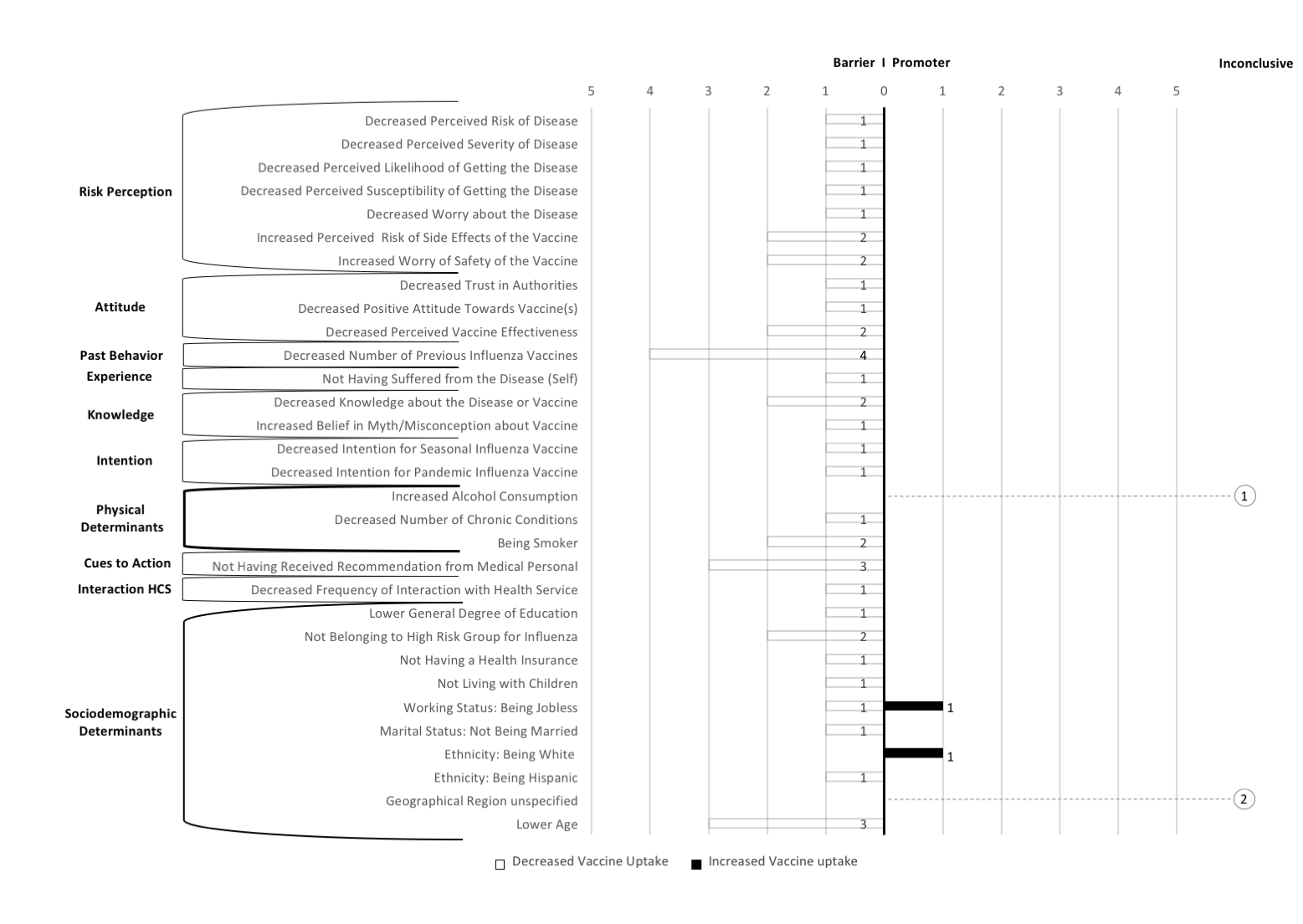

Supplement: S5 Fig — The figure visualizes the total numbers of studies reporting the variable as either decreasing (white) or increasing (black) vaccine acceptance or inconclusive (circled number). (TIFF) [file pone.0170550.s006.tiff]

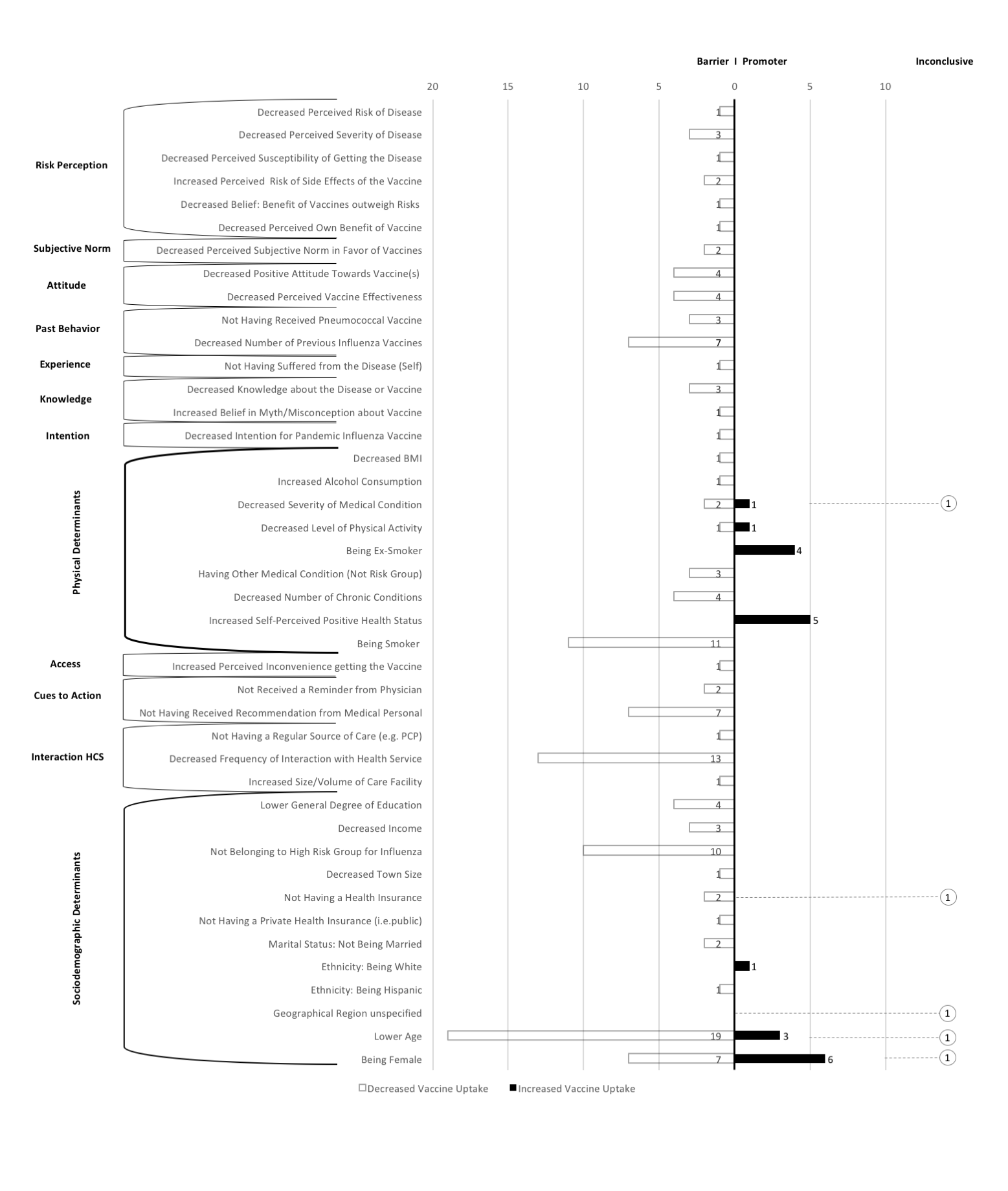

Supplement: S6 Fig — The figure visualizes the total numbers of studies reporting the variable as either decreasing (white) or increasing (black) vaccine acceptance or inconclusive (circled number). (TIFF) [file pone.0170550.s007.tiff]

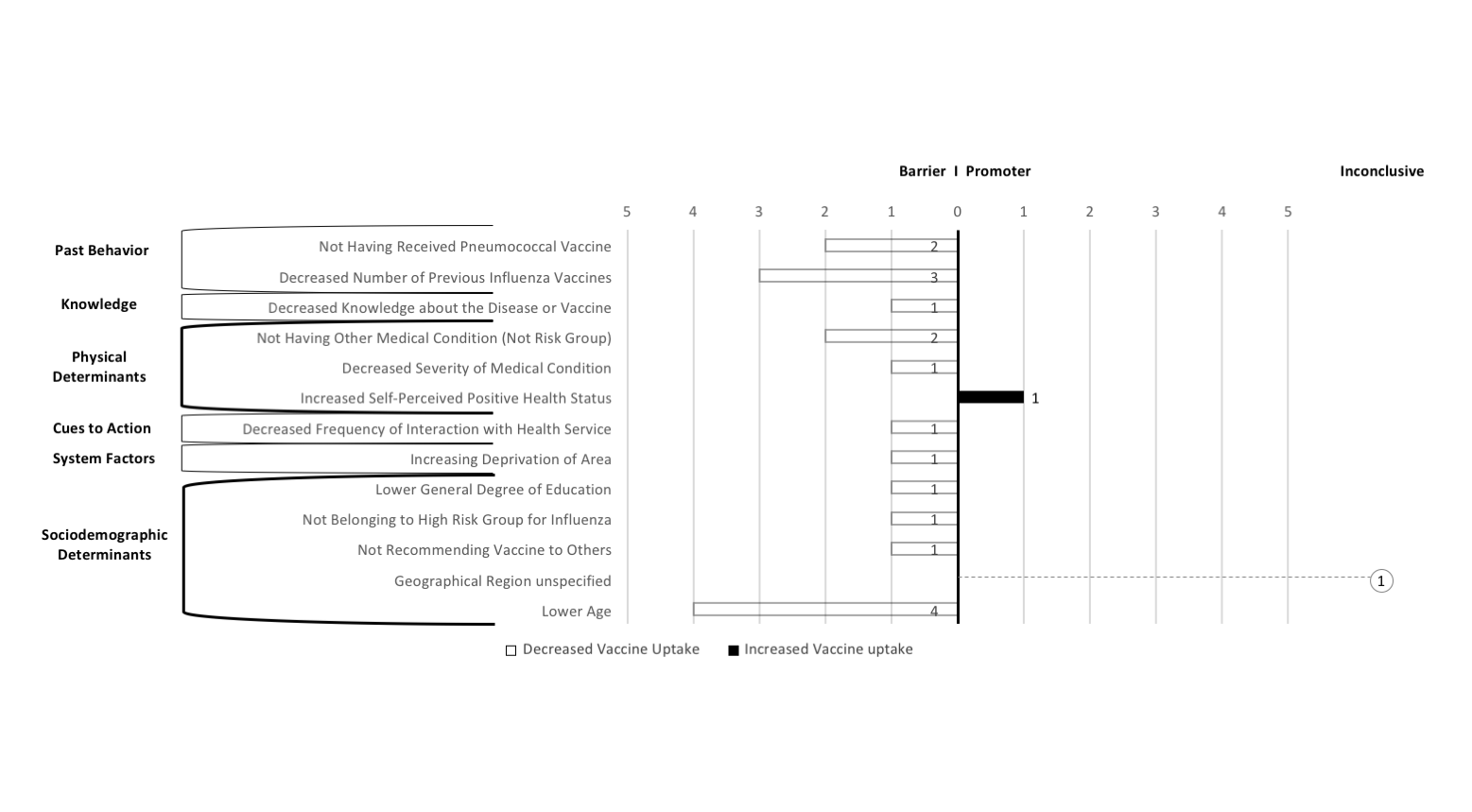

Supplement: S7 Fig — The figure visualizes the total numbers of studies reporting the variable as either decreasing (white) or increasing (black) vaccine acceptance or inconclusive (circled number). (TIFF) [file pone.0170550.s008.tiff]

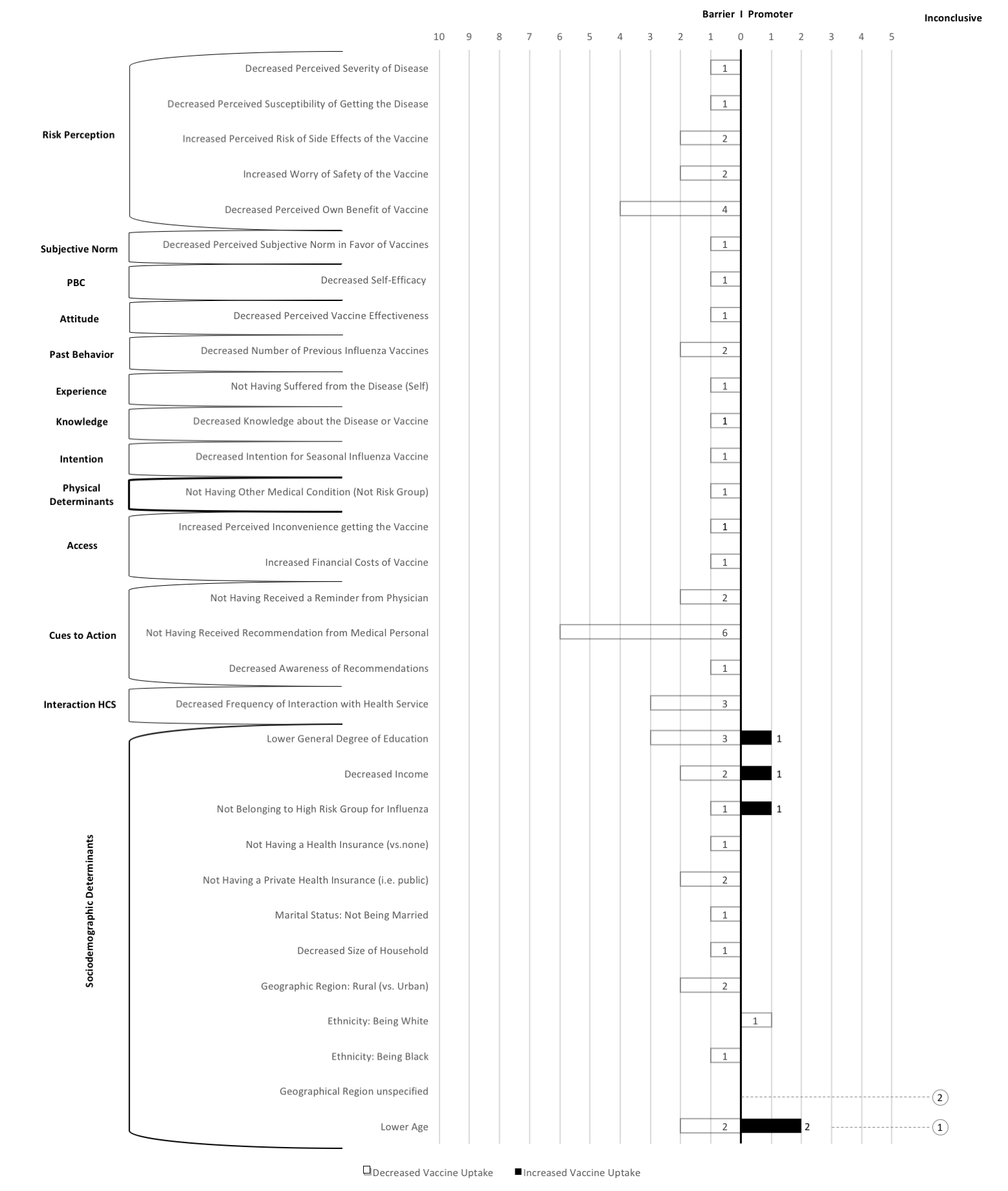

Supplement: S8 Fig — The figure visualizes the total numbers of studies reporting the variable as either decreasing (white) or increasing (black) vaccine acceptance or inconclusive (circled number). (TIFF) [file pone.0170550.s009.tiff]

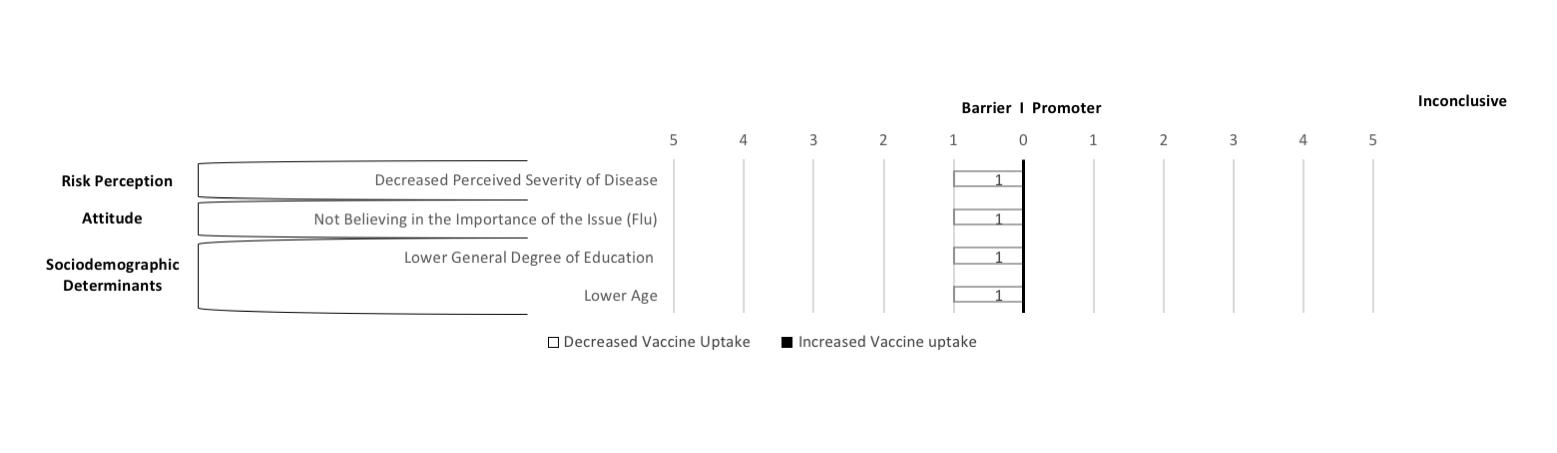

Supplement: S9 Fig — The figure visualizes the total numbers of studies reporting the variable as either decreasing (white) or increasing (black) vaccine acceptance or inconclusive (circled number). (TIFF) [file pone.0170550.s010.tiff]

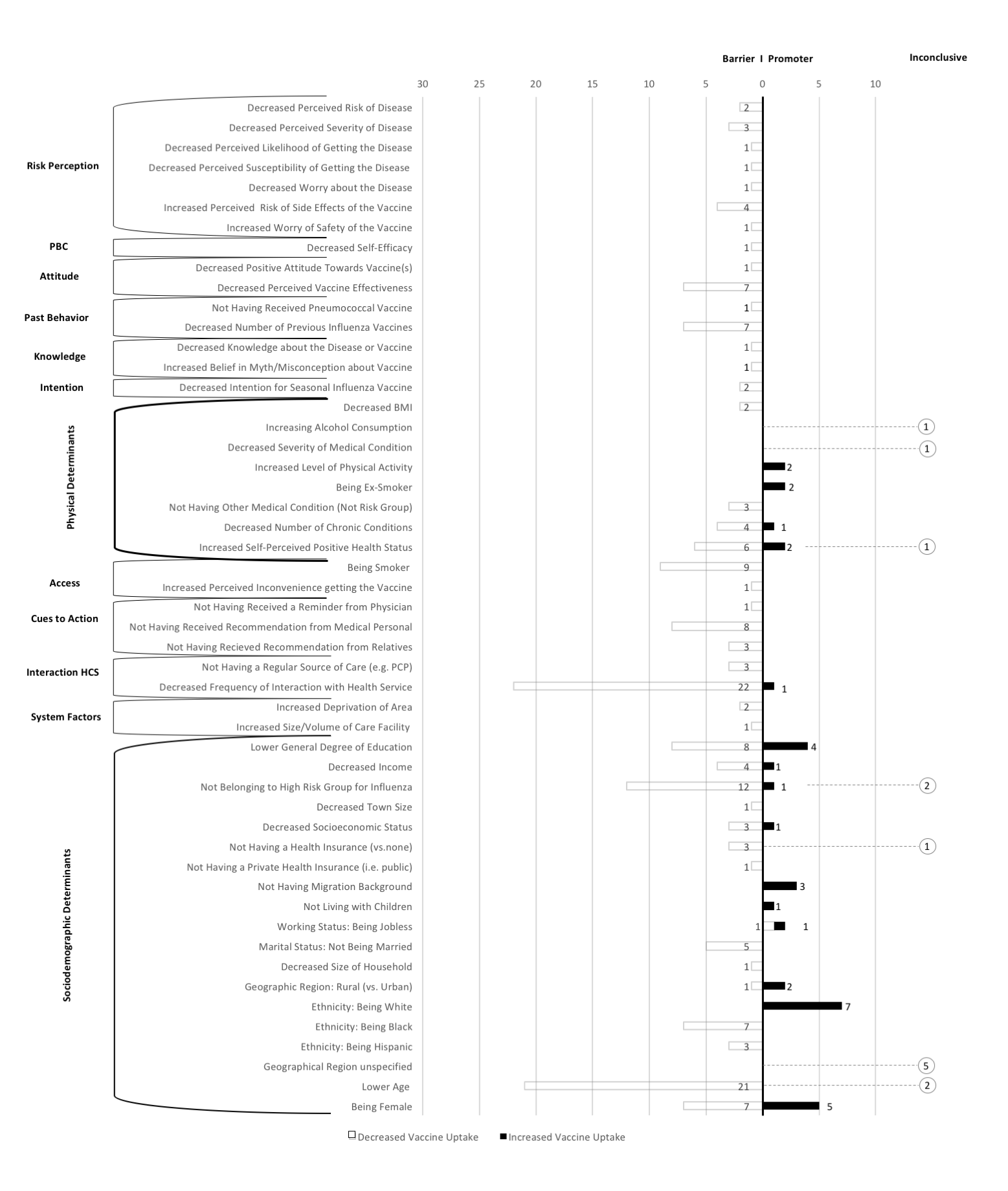

Supplement: S10 Fig — The figure visualizes the total numbers of studies reporting the variable as either decreasing (white) or increasing (black) vaccine acceptance or inconclusive (circled number). (TIFF) [file pone.0170550.s011.tiff]

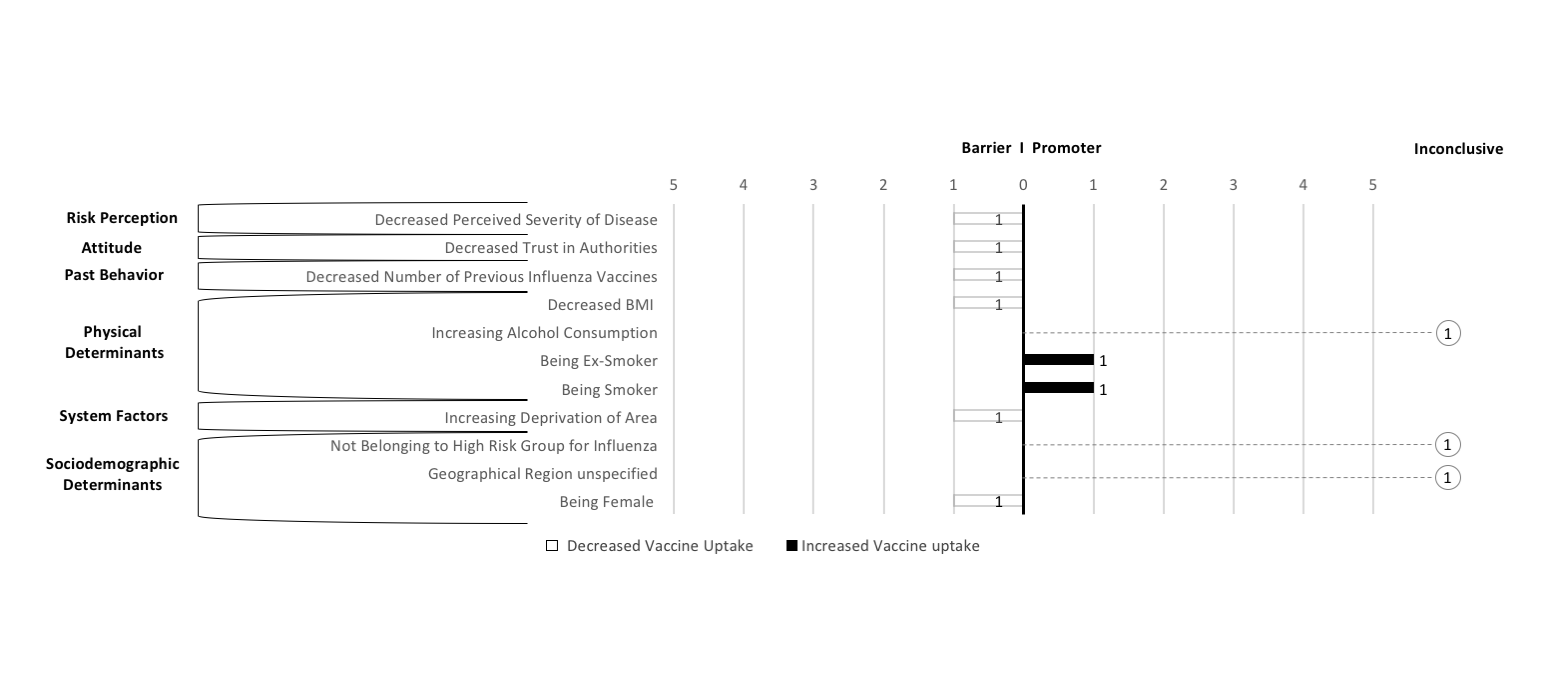

Supplement: S11 Fig — The figure visualizes the total numbers of studies reporting the variable as either decreasing (white) or increasing (black) vaccine acceptance or inconclusive (circled number). (TIFF) [file pone.0170550.s012.tiff]

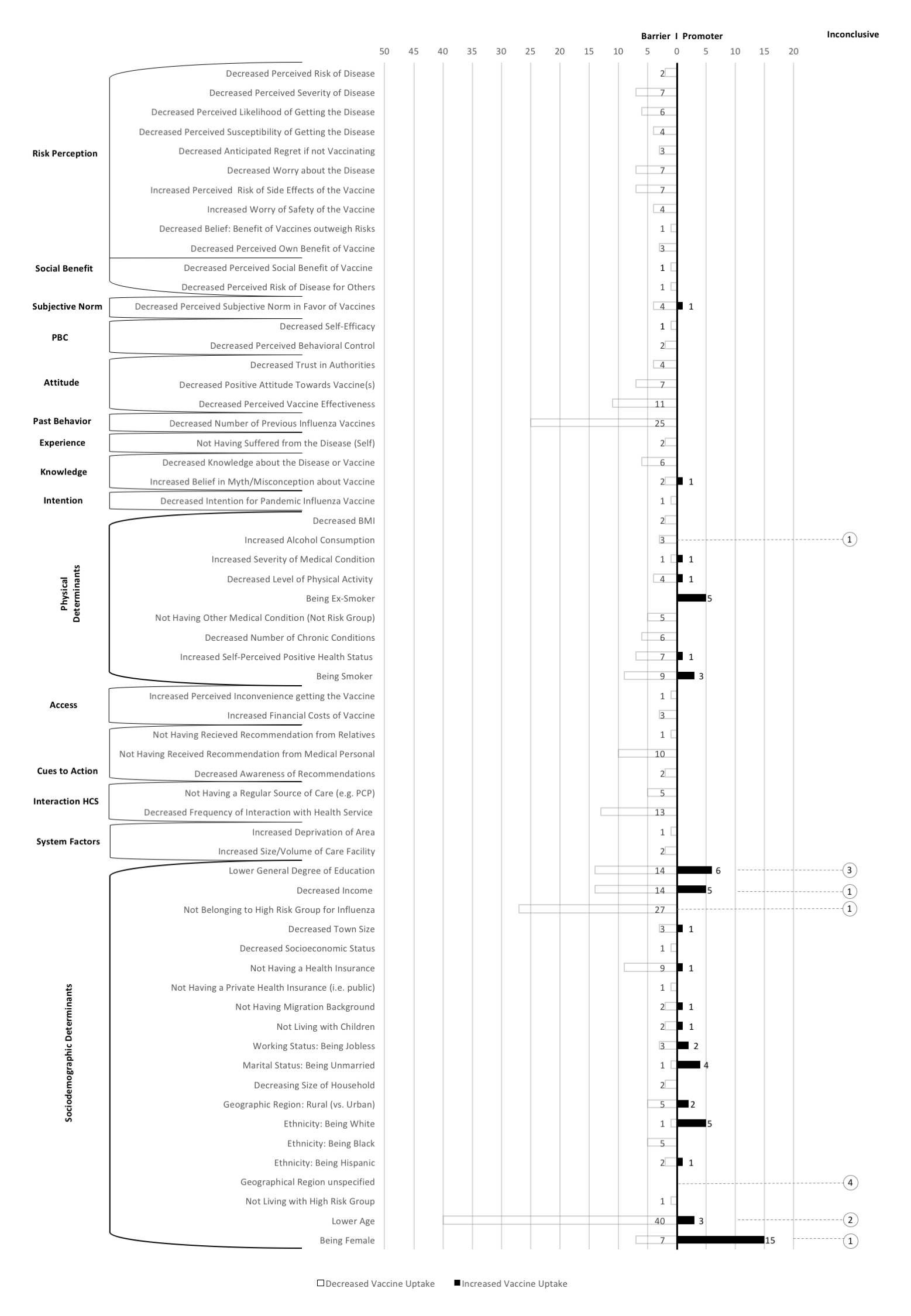

Supplement: S12 Fig — The figure visualizes the total numbers of studies reporting the variable as either decreasing (white) or increasing (black) vaccine acceptance or inconclusive (circled number). (TIFF) [file pone.0170550.s013.tiff]

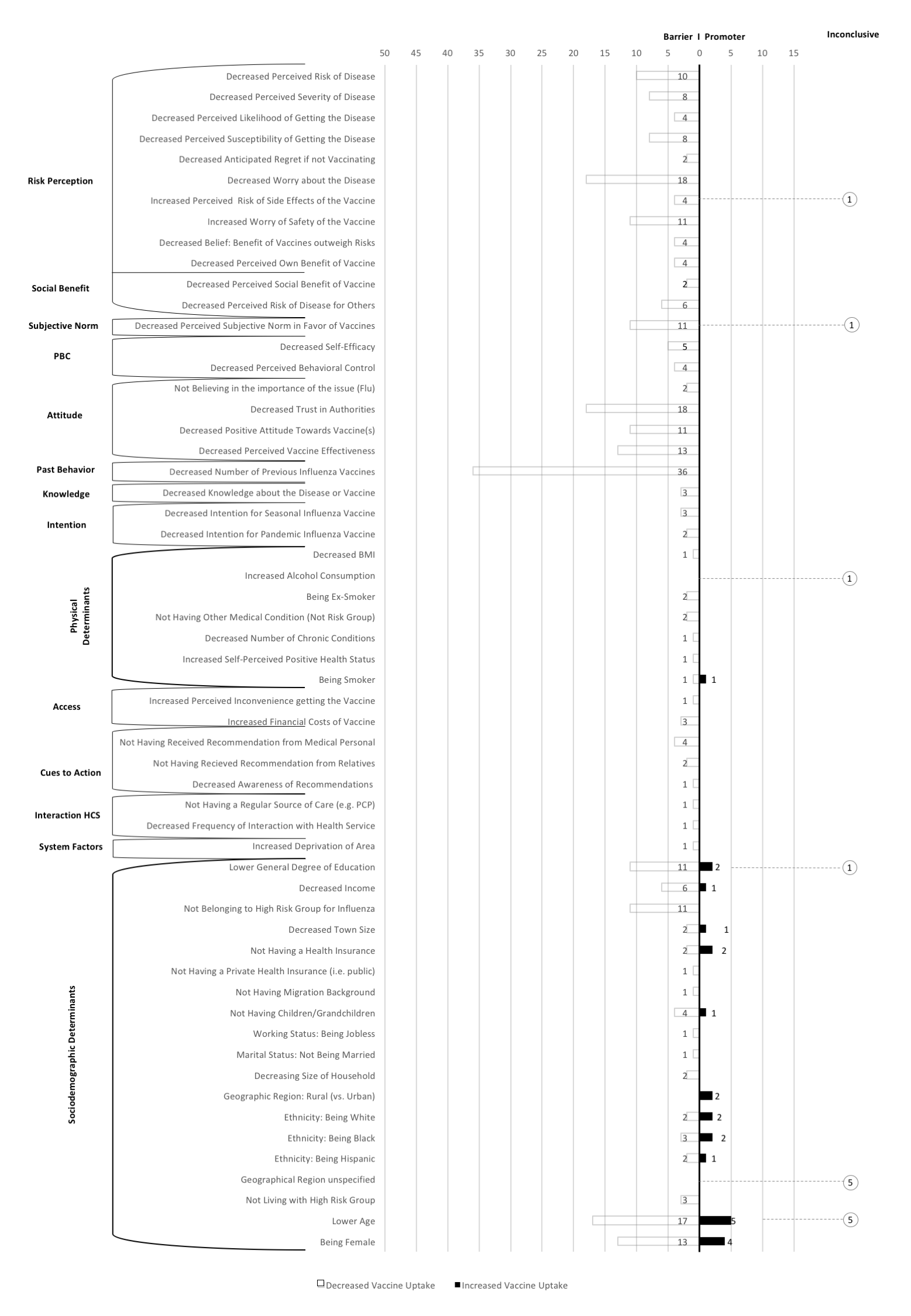

Supplement: S13 Fig — The figure visualizes the total numbers of studies reporting the variable as either decreasing (white) or increasing (black) vaccine acceptance or inconclusive (circled number). (TIFF) [file pone.0170550.s014.tiff]
