# Supplementary material for: Barriers of Influenza Vaccination Intention and Behavior – A Systematic Review of Influenza Vaccine Hesitancy, 2005 – 2016
Source: PLoS One. 2017 Jan 26;12(1):e0170550. doi: 10.1371/journal.pone.0170550 (PMC5268454; doi:10.1371/journal.pone.0170550)
Supplement: S2 Table — (PDF) [file pone.0170550.s015.pdf]

Risk Perception (Cognitive)

Perceived Risk of Influenza [1]	
How risky do you judge an infection with influenza to be?	**Response Option:** Visual Analog Scale (0 = not risky at all, 100 = very risky)
Perceived Probability of getting Influenza [2]	
What is the probability that you will get the flu this year if you do not get vaccinated?	**Response Option:** 7 point scale (1 = almost zero, 2 = very small, 3 = small, 4 = moderate, 5 = large, 6 = very large, 7 = almost certain)
Perceived Severity of Influenza [1]	
How severe do you judge an infection with influenza to be?	**Response Option:** 7 point scale (1 = not severe; 7 = very severe)
Perceived Susceptibility towards Influenza [3]	
I get the flu more easily than other people my age.	**Response Option:** 5 point scale (1 = strongly disagree; 5 = strongly agree)
Perceived Risk of Vaccine Adverse Events (VAE) [1]	
How risky do you judge the vaccination against influenza to be?	**Response Option:** Visual Analog Scale (0 = not risky at all, 100 = very risky)
Perceived Probability of VAE [1]	
What is the probability of experiencing adverse events if you get vaccinated against influenza?	**Response Option:** 7 point scale (1 = almost zero, 2 = very small, 3 = small, 4 = moderate, 5 = large, 6 = very large, 7 = almost certain)
Perceived Severity of VAE [1]	
How severe do you judge the possible adverse events of the vaccination against influenza to be?	**Response Option:** 7 point scale (1 = not severe; 7 = very severe)
Perceived Susceptibility towards VAE [3]	
I experience adverse events more easily than other people my age.	**Response Option:** 5 point scale (1 = strongly disagree; 5 = strongly agree)

Risk Perception (Affective)

Worry of Disease [4]	
How much do you worry about getting the flu this season?	**Response Option:** 5 point scale (1 = not at all; 5 = a lot)

Anticipated regret if not vaccinated [4]<br><br>*If I don't get a flu shot and end up getting the flu, I'd be mad at myself for not getting the shot.*	**Response Option:** 5 point scale (1 = strongly disagree; 5 = strongly agree)
Worry of VAE [4]<br><br>*How much do you worry about getting the influenza vaccine this season?*	**Response Option:** 5 point scale (1 =not at all; 5 = a lot)
Anticipated regret if vaccinated [4]<br><br>*If I get a flu shot and end up experiencing adverse events, I'd be mad at myself for getting the shot.*	**Response Option:** 5 point scale (1 = strongly disagree; 5 = strongly agree)
**Subjective Norms** [7,5]<br><br>*Most people who are important to me think that I should get vaccinated against influenza.*<br><br>*It is expected of me that I will get vaccinated against influenza.*<br><br>*The people in my life whose opinions I value would want me to get*<br><br>*vaccinated against influenza.*	**Response Option:** 7 point scale (1 = I totally disagree; 7 = I totally agree)<br><br>**Measure:** mean of sum score
**Perceived Behavioral Control** [7,5]<br><br>*For me, vaccinating against Influenza is possible.*<br><br>*If I wanted to get vaccinated against influenza in the next 6 months it would be easy.*<br><br>*How much control do you have over the decision to get vaccinated against influenza?*<br><br>*It is mostly up to me whether or not I get vaccinated against influenza.*<br><br>*The cost of the vaccine is a barrier to getting vaccinated against influenza.*	**Response Option:** 7 point scale (1 = I totally disagree; 7 = I totally agree)<br><br>**Measure:** mean of sum score
**Attitude** [7,5]<br><br>*Vaccinating against influenza is necessary.*<br><br>*Vaccinating against influenza is a good idea.*<br><br>*Vaccinating against influenza is beneficial.*	**Response Option:**7 point scale (1 = I totally disagree; 7 = I totally agree)<br><br>**Measure:** mean of sum score
**Knowledge** [6]	

HCPs are less susceptible to influenza infections than other people (false)<br>*Influenza is transmitted primarily by coughing and sneezing (true)*<br>*Influenza is more serious than a "bad cold" (true)*<br>*The signs and symptoms of influenza include fever, headache, sore throat, cough, nasal congestion, and aches and pains (true)*<br>*HCPs can spread influenza even when they are feeling well (true)*<br>*People with influenza can transmit the infection only after their symptoms appear (false)*<br>*Influenza is transmitted primarily by contact with blood and body fluids (false)*<br>*Influenza vaccination may not work if the vaccine contains the wrong mix of viruses (true)*<br>*The flu shot contains live viruses that may cause some people to get influenza (false)*<br>*Influenza vaccination does not work in some persons, even if the vaccine has the right mix of viruses (true)*<br>*Adults with influenza commonly experience nausea and vomiting or diarrhea (false)*<br>*Nasal spray influenza vaccine contains live flu viruses that may cause some people to get influenza (false)*<br>*Symptoms typically appear 8 to 10 days after a person is exposed to influenza (false)*	**Response Option:** yes; no; not sure<br><br>**Measure:** Percentage of correct answers.
**Experience with Influenza**	
Did you get influenza in the past?	**Response Option:** yes; no; not sure
**Experience with VAE**	
Did you experience adverse events after getting a flu shot?	**Response Option:** yes; no; not sure
**Past Behavior** [7]	
In the previous season, I have received the influenza vaccine.	**Response Option:** true; false
**Intention** [1]	
If you had the possibility to get vaccinated in the next week, what would you do?	**Response Option:** 7 point scale (1 = I would definitely not get vaccinated, 7 = I would definitely get vaccinated)

Note: Measurements are adapted to the issue of influenza where necessary.

[1] Betsch C, Haase N, Renkewitz F, Schmid P. The narrative bias revisited: What drives the biasing influence of narrative information on risk perceptions? Judgm Decis Mak 2015;10:241–64.

[2] Brewer NT, Chapman GB, Gibbons FX, Gerrard M, McCaul KD, Weinstein ND. Meta-analysis of the

relationship between risk perception and health behavior: The example of vaccination. Heal Psychol 2007;26:136–45.
[3] Nexøe J, Kragstrup J, Søgaard J. Decision on influenza vaccination among the elderly: A questionnaire study based on the Health Belief Model and the Multidimensional Locus of Control Theory. Scand J Prim Health Care 1999;17:105–10.
[4] Weinstein ND, Kwitel A, McCaul KD, Magnan RE, Gerrard M, Gibbons FX. Risk perceptions: Assessment and relationship to influenza vaccination. Heal Psychol 2007;26:146–51.
[5] Askelson NM, Campo S, Lowe JB, Smith S, Dennis LK, Andsager J. Using the theory of planned behavior to predict mothers' intentions to vaccinate their daughters against HPV. J Sch Nurs 2010;26:194–202. doi:10.1177/1059840510366022.
[6] Ofstead CL, Tucker SJ, Beebe TJ, Poland GA. Influenza vaccination among registered nurses: Information receipt, knowledge, and decision-making at an institution with a multifaceted educational program. Infect Control Hosp Epidemiol 2008;29:99–106.
[7] Ajzen I. The theory of planned behavior. Organ Behav Hum Decis Process 1991;50:179–211. doi:10.1016/0749-5978(91)90020-T.
